# Chemokine-receptor-dependent inflammatory responses contribute to two-hit-induced experimental necrotizing enterocolitis and represent a potential therapeutic target

**DOI:** 10.3389/fimmu.2026.1830078

**Published:** 2026-07-15

**Authors:** Saravanan Subramanian, Nazeer Hussain Khan, Hua Geng, Devarsh Rupeshkumar Shah, Heng-Fu Bu, Qianming Jiang, Pauline M. Chou, Xiao Wang, Joann Romano-Keeler, Isabelle G. De Plaen, Xiao-Di Tan

**Affiliations:** 1 Pediatric Mucosal Inflammation and Regeneration Research Program, Center for Pediatric Translational Research and Education, Department of Pediatrics, College of Medicine, University of Illinois at Chicago, Chicago, IL, United States; 2 Department of Pathology, Feinberg School of Medicine, Northwestern University, Chicago, IL, United States; 3 Division of Neonatology, Department of Pediatrics, College of Medicine, University of Illinois at Chicago, Chicago, IL, United States; 4 Division of Neonatology, Department of Pediatrics, Feinberg School of Medicine, Northwestern University, Chicago, IL, United States

**Keywords:** breastmilk feeding and formula feeding, chemokine-receptor signaling, dysbiosis, intestinal inflammation, necrotizing enterocolitis

## Abstract

**Background:**

Necrotizing enterocolitis (NEC) is a severe multifactorial inflammatory disorder of the preterm intestine for which effective preventive and therapeutic strategies remain limited. Using formula feeding (FF)-associated dysbiosis combined with subsequent viral inflammation, we previously demonstrated that this clinically relevant two-hit strategy induces NEC through a complex mechanism involving the *Klebsiella oxytoca*-derived metabolite tilivalline (TV) and NK1.1**^+^** cells. Here, we investigated whether chemokine-receptor-dependent inflammatory responses associated with inflammatory cell recruitment contribute to NEC pathogenesis and whether pharmacologic blockade of chemokine-receptor signaling attenuates disease progression.

**Methods:**

NEC was induced in C57BL/6 mouse pups using two novel and clinically relevant two-hit models: FF combined with R837-triggered viral inflammation, or TV gavage in dam-fed pups followed by R837 treatment. In some experiments, pups were pretreated subcutaneously with TAK-779, a pharmacologic inhibitor of CCR2/CCR5/CXCR3 signaling. NEC-like intestinal injury was assessed by intestinal permeability and histological analysis. RT-qPCR and flow cytometry were used to evaluate chemokine-receptor expression and inflammatory cell infiltration, respectively. A human NEC RNA-seq dataset (E-MTAB-15683) was used to evaluate the expression of chemokine-receptor genes.

**Results:**

Following the R837-induced second hit, mouse pups previously subjected to the FF-associated first hit exhibited robust induction of chemokines and chemokine receptors, accompanied by recruitment of inflammatory cells and the development of NEC. TAK-779 markedly reduced the expression of chemokines and their receptors, attenuated inflammatory cell recruitment, and protected against NEC induced by the sequential FF- and viral inflammation-associated two-hit process. Similarly, subjecting BF pups to TV (first hit) followed by R837 treatment (second hit) resulted in heightened expression of chemokines and their receptors, accompanied by NEC development; both effects were prevented by TAK−779 pretreatment. Notably, analysis of human NEC transcriptomic data revealed increased expression of multiple chemokines and chemokine receptors in diseased intestinal tissues.

**Conclusions:**

Our findings demonstrate that chemokine-receptor-dependent inflammatory responses contribute to the propagation of NEC by promoting the accumulation of NK1.1^+^ cells and other inflammatory cells. Pharmacologic inhibition of chemokine-receptor signaling attenuates NEC in clinically relevant experimental two-hit models that recapitulate key features of disease pathogenesis, highlighting these pathways as potential therapeutic targets warranting further investigation.

## Graphical Abstract

**Figure:**
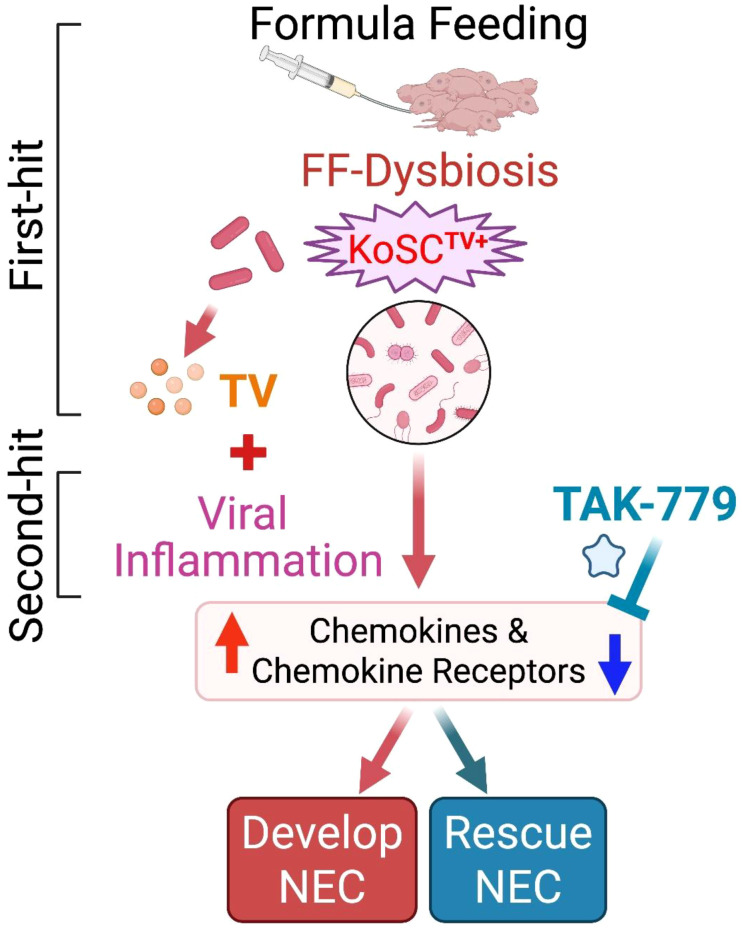

## 1 Introduction

Necrotizing enterocolitis (NEC) is a devastating inflammatory disease that primarily affects the immature distal small intestine (ileum) of preterm infants (1, 2). NEC affects up to 11% of very low birth weight infants (<1500 g) each year in the United States (3). It is characterized by segmental mucosal necrosis, microbial overgrowth, pneumatosis intestinalis, and massive infiltration of inflammatory cells, often leading to sepsis, multiorgan failure, and death (4, 5). Despite decades of intensive research, NEC remains a major clinical challenge with no effective targeted therapies, mainly because its underlying mechanisms are not fully understood. NEC is widely recognized as a multifactorial disorder arising from the convergence of several predisposing factors, including prematurity, formula feeding (FF), microbial dysbiosis, hypoxia-ischemia, and aberrant immune activation (6, 7). NEC is increasingly recognized as developing through a multi-hit process in which early-life predisposing factors create a vulnerable intestinal environment that is subsequently exacerbated by secondary inflammatory or infectious insults. Although bacterial colonization and aberrant immune activation have received considerable attention in NEC pathogenesis, emerging evidence suggests that viral infections with gastrointestinal tropism can further exacerbate mucosal inflammation and tissue injury in the neonatal gut (8–11). Kaelin et al. revealed that alterations in bacterial-viral interactions preceded the sudden onset of NEC (12). However, how early-life feeding-associated microbial factors serve as an initial priming hit and interact with a subsequent viral inflammatory second hit to disrupt intestinal homeostasis in the developing gut remains poorly understood.

Breastmilk feeding (BF) is known to confer protection against NEC, whereas FF is a major risk factor for disease onset (13). Human milk promotes colonization of beneficial commensal microbiota, supports immune maturation, and delivers bioactive factors that sustain mucosal tolerance (14, 15). In contrast, FF alters the gut microbial composition, favoring the colonization of distinct microbial communities that differ markedly from those in breastmilk-fed infants (16, 17). FF-associated dysbiosis correlates with increased intestinal inflammation and higher NEC incidence (5, 18). Importantly, FF alone is generally insufficient to induce NEC. Clinical studies showed that NEC-associated bacteria, such as *Klebsiella oxytoca*, are present in the biofilm of neonatal nasogastric enteral feeding tubes (19, 20). Thus, preferential colonization of the gut with these bacteria following formula gavage is considered a predisposing factor that constitutes a clinically relevant first hit, priming the neonatal intestine for injury following a subsequent inflammatory second hit. Indeed, our previous work demonstrated that FF-associated microbial signals serve as a predisposing factor that, together with a subsequent viral inflammation-associated second hit, drives NEC development through natural killer (NK1.1^+^) cell-dependent mechanisms (21). In this context, we identified *Klebsiella oxytoca* strains enriched in FF-fed neonates that produce tilivalline (TV), a genotoxic pyrrolobenzodiazepine metabolite (22, 23). Notably, bacterial strains of TV-producing *Klebsiella oxytoca* species complex (KoSC^TV+^) are frequently detected in NEC patients (24–26). These *til*^+^ *Klebsiella* strains synthesize tilimycin, which is further converted into TV (22). The luminal ratio of these metabolites varies across the murine gastrointestinal tract, with TV emerging as the predominant metabolite in the small intestine (27). Notably, TV acts as a first hit that predisposes the intestine to NEC development in response to a subsequent viral inflammatory challenge through an NK1.1^+^ cell-dependent mechanism (21), thereby contributing to the pathogenesis of NEC. However, the mechanisms linking the two-hit model to inflammatory cell recruitment and intestinal injury remain unknown.

To address this gap, we hypothesized that chemokine-receptor-dependent inflammatory responses represent a mechanistic link through which the sequential effect of FF or TV, followed by subsequent viral inflammation, contributes to NEC development. To test this hypothesis, we first examined the expression of chemokines (*Ccl2, Ccl3, Ccl4, Ccl5, Cxcl1, Cxcl2, Cxcl9, Cxcl10*, and *Cxcl11*) and chemokine receptors (*Ccr2, Ccr5, Cxcr2*, and *Cxcr3*) in the ileum of BF, FF, and human donor milk-fed (HMF) neonatal mice following viral inflammation induced by R837, a Toll-like receptor 7 [TLR7] agonist. Second, we examined whether TV, as an FF-associated microbial metabolite, similarly modulates the expression of these chemokines and receptors to potentiate immune activation during viral inflammation. Third, we tested whether pharmacological blockade of CCR2/CCR5/CXCR3 signaling using TAK-779 (28) attenuates NEC development in both experimental two-hit models. Finally, we reanalyzed a recently published RNA-sequencing (RNA-seq) dataset to determine whether chemokine-receptor signaling components identified in our experimental models are similarly dysregulated in human NEC. Our findings suggest that chemokine-receptor-dependent inflammatory responses constitute an important downstream inflammatory pathway through which the two-hit process promotes NEC development. Targeting this inflammatory event may represent a promising therapeutic strategy for NEC prevention.

## 2 Materials and methods

### 2.1 Animals

C57BL/6 (B6) wild-type (WT) mice (stock no. 000664) were purchased from the Jackson Laboratory (Bar Harbor, ME). All mice were housed in a specific pathogen-free (SPF) barrier facility with ad libitum access to water and standard chow under a 12-hour light/dark cycle. Breeding pairs (8–12 weeks old) were used to generate neonatal pups, which remained with the dam until experimental manipulation. The mice were allowed to give birth naturally. Both male and female neonates were included in the experiments, and pups were age-matched across experimental groups. All mice used in the study were euthanized humanely with CO_2_ inhalation (3 L/min) followed by cervical dislocation, in accordance with the American Veterinary Medical Association (AVMA) Guidelines for the Euthanasia of Animals. All experimental procedures were approved by the Institutional Animal Care and Use Committee (IACUC) and conducted in accordance with all relevant ethical guidelines for the care and use of laboratory animals.

### 2.2 Postnatal feeding intervention

Both male and female mouse pups were included in this study. Briefly, mouse pups (WT) were subjected to feeding interventions at postnatal day 7 (P7) without prior knowledge of their gender features. To minimize litter-specific effects, experimental groups were generated using pups derived from multiple independent litters, and pups were randomly assigned to the appropriate experimental groups. No individual litter contributed exclusively to a single experimental group. For breastmilk feeding (BF) groups, the pups remained with the dam throughout the study period and were monitored daily. Two alternative feeding strategies were used in this study, including human donor milk-feeding (HMF) and formula feeding (FF). Neonatal pups assigned to HMF or FF were separated from the dam at P7 and placed in a 37 °C humidified neonatal incubator (Air-Shield Vickers Medical, Hatboro, PA) for the duration of the experiments. The room for maintaining HMF or FF pups was on a 12:12 h light-dark cycle. The HMF group served as a control to assess the impact of confounding factors related to gavage feeding. The neonatal pups (P7) were gavage fed with either human donor milk (200 mL/kg/day) or Esbilac formula (Esbilac, 200 mL/kg/day) every 4 hours for 4 days (P7 – P11). Feed volumes started at 0.1 ml every 4 hours beginning at P7 and gradually increased to 0.14 ml per feeding by P11 to accommodate normal growth. The pups subjected to alternative feedings were monitored every 3–6 hours throughout the study.

In this study, pups were excluded if they were sick or had a small litter size (n ≤ 2 pups/litter). If the pups had discomfort or were injured in the trachea while gavage feeding, they were also excluded from this study. At the end of the experiments, we did not confirm the gender characteristics of the pups. Sex determination was not performed because reliable sex identification is challenging during the early neonatal period used in this study. Consequently, both male and female pups were included, but sex-specific analyses could not be performed. Therefore, potential sex-dependent differences in immune and inflammatory responses cannot be excluded and should be considered when interpreting the findings. Mouse pups were humanely euthanized as described above at the end of the experiments. The serum and intestinal tissues were collected and processed for further studies (18, 21).

### 2.3 Administration of tilivalline

BF mouse pups (P7) were orally gavaged with TV (20 μg/pup per feeding, twice daily [b.i.d]; Santa Cruz Biotechnology) for 3 days from P7 – P10. The selected TV dose was based on our previous studies demonstrating reproducible enhancement of NEC susceptibility without inducing overt intestinal injury in the absence of an inflammatory stimulus (21). An equal volume of DMSO was administered to the pups as the solvent control.

### 2.4 Induction of viral inflammation in the gut with TLR7 agonist

The pups were orally gavaged with R837 (25 μg/gm body weight; InvivoGen), a Toll-like receptor 7 (TLR7) agonist, or vehicle control at P10. R837 was dissolved in sterile endotoxin-free water. Within the experimental two-hit models, R837 administration served as the secondary inflammatory stimulus (second hit) following the initial hit caused by priming the gut with formula feeding or tilivalline exposure. Pups were monitored at 3-h intervals throughout the experiment. Pups were humanely euthanized as described above at 24 h after treatment. The intestinal tissues were collected and processed for further studies.

### 2.5 Two-hit models of experimental NEC

Two approaches were employed using our previously described protocols (21): (1) Mouse pups were subjected to FF from P7 to P11 to establish a priming first-hit condition characterized by formula feeding-associated dysbiosis. Pups were then gavaged with R837 at P10 to model a viral inflammation-associated second hit. (2) Alternatively, dam-fed mouse pups were gavaged with TV from P7 to P10 to establish a microbial priming first-hit condition and were subsequently challenged with R837 at P10 as the viral inflammation-associated second hit. Pups were euthanized 24 h after R837 administration in both experimental two-hit models. Importantly, these models were selected to investigate a defined inflammatory mechanism within clinically relevant two-hit paradigms rather than to recapitulate every aspect of NEC pathogenesis.

### 2.6 Administration of TAK-779

In some experiments, to determine whether inhibition of chemokine-receptor signaling attenuated inflammatory responses and disease severity in the experimental two-hit models, FF or BF mouse pups (P7) were subcutaneously pretreated with TAK-779 (2.5 µg/gm, b.i.d; Sigma) for 3 days (P7-P10) to block CCR2, CCR5, and CXCR3 signaling. Pups were then subjected to R837 treatment to induce the viral inflammation-associated second hit, as described above.

### 2.7 Monitoring body weight changes and survival

Based on experimental designs, all pups were weighed and divided into appropriate experimental groups. The baseline weights of the pups did not differ significantly among experimental groups. The pups were monitored closely and weighed daily throughout the study period. Survival was recorded at 3-hour intervals or daily, depending on the experiment.

### 2.8 Measurement of intestinal permeability

Mouse pups underwent a 3-hour fasting period to evaluate the gut barrier function at the end of the experiments. Following fasting, the pups were administered fluorescein isothiocyanate (FITC)-dextran (FD-10; 400 mg/kg; Sigma) by gavage as described in our previous publications (2, 18, 21). Four hours later, the mouse pups were humanely euthanized, and serum samples were collected for the quantification of FD-10 levels. The FITC fluorescence signal was measured in serum samples using a SpectraMax iD3 multi-mode reader (Molecular Devices, LLC, San Jose, CA) equipped with excitation at 485 nm and emission at 528 nm wavelengths.

### 2.9 Histological examination

Small intestines were collected and fixed overnight in 10% buffered formalin and then embedded in paraffin. Tissue sections (4 μm) were stained with hematoxylin-eosin (H&E) for histological examination. H&E-stained slides were examined under a light microscope. Intestinal injury was graded in a blinded fashion on a 5-point scale using a histological scoring system as described in our previous publications (18, 21). Grade 0: no injury; Grade 1: injury limited to the tip of the villi; Grade 2: mid-villous disruption and moderate lamina propria (LP) separation; Grade 3: complete villous disruption and edema in submucosa; Grade 4: transmural injury. Histological intestinal injury was evaluated across multiple segments throughout the tissue samples on each slide. The segment with the most severe injury was used as the representative injury score. This approach was selected to quantify the most severe focal NEC-like lesions, which are considered clinically relevant, but may emphasize peak injury rather than overall severity across the entire intestinal section. For presentation, we acquired images using the Leica Thunder Microscope Imager System equipped with a DMC2900 Color Camera (Wetzlar, Germany), followed by processing and assembling with Adobe Photoshop 2021 (San Jose, CA).

### 2.10 Quantitative reverse-transcription PCR

Ileum tissue samples were collected, directly snap-frozen, and stored at −80 °C for RT-qPCR studies. Total RNA was extracted from the tissue samples using the TRIzol reagent (Life Technologies) according to the manufacturer’s instructions. RNA purity and concentration were measured with a NanoDrop spectrophotometer (Agilent Technologies, Santa Clara, CA). First-strand cDNA was synthesized using the iScript cDNA synthesis kit (Bio-Rad). PCR amplification was performed using PowerUp SYBR Green Master Mix (Thermo Fisher Scientific) according to the manufacturer’s instructions on the QuantStudio™ 6 real-time PCR system (Thermo Fisher Scientific). The primers for RT-qPCR were obtained from Integrated DNA Technologies (IDT), and the sequences for target genes are shown in **Table 1**. The relative expression of target genes was estimated and normalized to the expression of the housekeeping gene glyceraldehyde-3-phosphate dehydrogenase (*Gapdh*). The relative expression levels of target genes were reported as fold induction using the 2^–ΔΔCT^ method.

**Table 1:** Primer sequences used for RT-qPCR.

Target gene	Forward sequence (5’-3’)	Reverse sequence (5’-3’)
*Ccl2*	AGGTCCCTGTCATGCTTCTG	TCTGGACCCATTCCTTCTTG
*Ccl3*	TGAAACCAGCAGCCTTTGCTC	AGGCATTCAGTTCCAGGTCAGTG
*Ccl4*	AAACCTAACCCCGAGCAACA	CCATTGGTGCTGAGAACCCT
*Ccl5*	ACACCACTCCCTGCTGCTTT	GACTGCAAGATTGGAGCACTTG
*Cxcl1*	GACCATGGCTGGGATTCACC	CCAAGGGAGCTTCAGGGTCA
*Cxcl2*	CGCCCAGACAGAAGTCATAG	TCCTCCTTTCCAGGTCAGTTA
*Cxcl9*	TCTGCCATGAAGTCCGCTG	CAGGAGCATCGTGCATTCCT
*Cxcl10*	TGCTGGGTCTGAGTGGGACT	TTTCTTTCAGGGACAGCCTGTT
*Cxcl11*	GGAAGGTCACAGCCATAGCC	GATCTCTGCCATTTTGACGGC
*Ccr2*	ACAGCTCAGGATTAACAGGGACTTG	ACCACTTGCATGCACACATGAC
*Ccr5*	AGGCCATGCAGGCAACAG	TCTCTCCAACAAAGGCATAGATGA
*Cxcr2*	CCTTGAATGCTACGGAGATT	AGGGTAGTAGAGGTGTTTGC
*Cxcr3*	CAGCCTGAACTTTGACAGAACCT	GCAGCCCCAGCAAGAAGA
*Gapdh*	AACTTTGGCATTGTGGAAGG	ACACATTGGGGGTAGGAACA

### 2.11 Flow cytometry

FACS analysis was performed in the ileum tissue samples of FF pups with or without TAK-779 and R837 treatment as described in our previous publications (2, 18, 21), to characterize the infiltration of immune cells following inhibition of CCR2, CCR5, and CXCR3 signaling. Briefly, the ileum samples were washed with phosphate-buffered saline (PBS) and cut longitudinally under a dissection microscope to carefully remove mesenteric vasculature and pancreatic tissues. The tissue samples were then treated with dissociation buffer [PBS containing 5 mmol/L EDTA, 15 mmol/L HEPES, 1 mmol/L dithiothreitol, and 10% fetal bovine serum (FBS)] for 30 min at 37 °C. After the supernatants were discarded, the tissues were washed in PBS and subjected to digestion buffer [Dulbecco’s modified Eagle’s medium (DMEM) containing 1 mg/mL collagenase VIII (Sigma), 15 mmol/L HEPES, 5 mmol/L CaCl_2_, and 2% FBS] for 15 min at 37 °C. Cells were filtered through a 40-μm strainer, centrifuged, washed, and enumerated, and the prepared cells were used for downstream analyses. The cell suspension was stained with the viability dye FVD-eFluor-506 (Thermo Fisher Scientific) in PBS for 15 min on ice, followed by Fc block (Miltenyi Biotec, San Diego, CA) in staining buffer containing PBS and 2% FBS for 15 min on ice. Cells were further incubated with the antibodies listed in **Table 2** in staining buffer for 20 min on ice, followed by cell fixation using 2% paraformaldehyde for 15 min. After fixation, FACS was performed on a Cytek Aurora full-spectrum flow cytometer (Cytek Biosciences, CA) and analyzed with FlowJo version 10.8.1 (FlowJo LLC, Ashland, OR).

**Table 2:** Antibodies used for flow cytometry.

Antigen	Clone	Fluorochrome	Concentration	Vendor
CD3	145-2C11	APC/Cy7	1:20	BD
CD64	X54-5/7.1	AF647	1:20	BD
MHC-II	M5/114.15.2	PE/Cy7	1:800	BioLegend
SigF	E50-2440	PE/CF594	1:20	BD
Ly6G	1A8	BV711	1:50	BD
CD45	30-F11	BV570	1:100	BioLegend
L/D	Fixable Viability Dye	eFluor 506	1:1000	eBioscience
Fc block	2.4G2	–	1:100	BD
Ly6C	HK1.4	BV421	1:200	BioLegend
CD11b	M1/70	BUV737	1:400	BD
CD19	1D3	BUV563	1:50	BD
CD11c	HL3	BUV395	1:20	BD
NK1.1	PK136	BV786	1:50	eBioscience
CD4	RM4-5	AF700	1:200	BD
CD8	53-6.7	BB700	1:100	BD

Note that the specificity of antibodies was verified based on information from datasheets provided by vendors.

To eliminate debris and clumped cells, cells were first gated for FSC-A vs. SSC-A based on size and granularity. Using FSC-A vs. FSC-H and SSC-A vs. SSC-H gates, single cells were obtained. These single cells were further sub-gated using the fixable live-dead viability dye to identify live cells. Live cells were further gated as leukocytes based on the pan-hematopoietic marker CD45. Live CD45+ cells were subsequently analyzed for immune-cell subsets as described in our previous publication (21). CD45^+^ cells were subjected to CD11b^+^ and CD11c^+^ gating. The CD11b^+^ cell populations were further gated to separate neutrophils (CD45^+^CD3^-^CD11b^+^Ly6g^+^), eosinophils (CD45^+^CD3^-^CD11b^+^SigF^+^), monocytes (CD45^+^CD3^-^CD11b^+^Ly6c^+^), macrophages (CD45^+^CD3^-^CD11b^+^ Ly6G^-^MHC-II^+^CD64^+^), dendritic cells (CD45^+^CD3^-^CD11b^+^Ly6G^-^CD64^-^CD11c^+^), and NK1.1^+^ cells (CD45^+^CD3^-^CD11b^+^NK1.1^+^). The CD11b^-^ and CD11c^-^ lymphocyte-enriched fraction was further gated for CD3^+^ and CD3^-^ cells. The CD3^-^ cells were gated for B cells (CD45^+^CD11b^-^CD3^-^CD19^+^). CD4^+^ T cells (CD45^+^CD11b^-^CD3^+^CD19^-^CD4^+^) and CD8 T cells (CD45^+^CD11b^-^CD3^+^CD19^-^CD8^+^) were further characterized from the lymphocyte-enriched fraction.

### 2.12 Meta-transcriptomic analysis of the human NEC patient’s database

RNA sequencing (RNA-seq) dataset E-MTAB-15683 was previously generated and deposited in the ArrayExpress database by Yang et al., an independent research group not associated with our research team (29). This dataset was obtained for secondary analysis. Briefly, the dataset contains an RNA-seq count matrix from intestinal tissue samples derived from 7 neonates with surgically confirmed NEC and 7 gestational age-matched controls diagnosed with congenital intestinal atresia. The count matrix was first normalized, and differentially expressed genes (DEGs) were identified using the DESeq2 package (version 1.46) in R software (version 4.4). Statistical significance was defined as an adjusted *P* value < 0.05. By analyzing the DEG profile, we examined whether chemokine-receptor signaling pathways identified in the experimental two-hit models were similarly dysregulated in human NEC tissues.

### 2.13 Statistical analysis

Statistical details of all experiments and data analyses are provided in this section, as well as in the figure legends and in the results section. All experiments were performed at least twice with duplicate samples. Statistical analysis was performed with GraphPad Prism 8 (GraphPad Software, San Diego, CA). Animal survival data were analyzed by the log-rank test. The non-parametric chi-square (χ^2^) test was used to compare the incidence of NEC severity (Grade ≥ 2). The statistical significance of the experimental group differences was analyzed with an independent Student’s t-test or one-way analysis of variance (ANOVA) followed by a Tukey post-test or interaction test. Data are presented as mean ± standard deviation (SD). A p-value <0.05 was considered statistically significant. For RT-qPCR analyses, each chemokine and chemokine receptor gene was analyzed individually using the statistical methods described above. Because these analyses were hypothesis-driven and focused on predefined target genes, no additional correction for multiple testing across the complete 13-gene panel was applied. Therefore, these findings should be interpreted with appropriate caution and validated in future studies. Statistical significance was denoted as ^∗^*p* < 0.05. ^∗∗^*p* < 0.01, ^∗∗∗^*p* < 0.001, ^∗∗∗∗^*p* < 0.0001.

## 3 Results

### 3.1 A two-hit process involving formula feeding and viral inflammation increases the expression of chemokines and chemokine receptors during NEC development

Chemokines and their receptors play a central role in orchestrating intestinal inflammatory responses and have emerged as important therapeutic targets for inflammatory bowel disease (28, 30–32). We previously demonstrated that formula-fed (FF) neonatal mice developed experimental NEC following viral inflammation through an NK1.1^+^ cell-dependent mechanism (21). Because our experimental model recapitulates a clinically relevant two-hit paradigm (21), in which FF serves as a sensitizing first hit and viral inflammation provides a subsequent second hit, we sought to investigate whether chemokine-receptor signaling represents an important inflammatory pathway associated with NEC progression.

**Figure 1:**
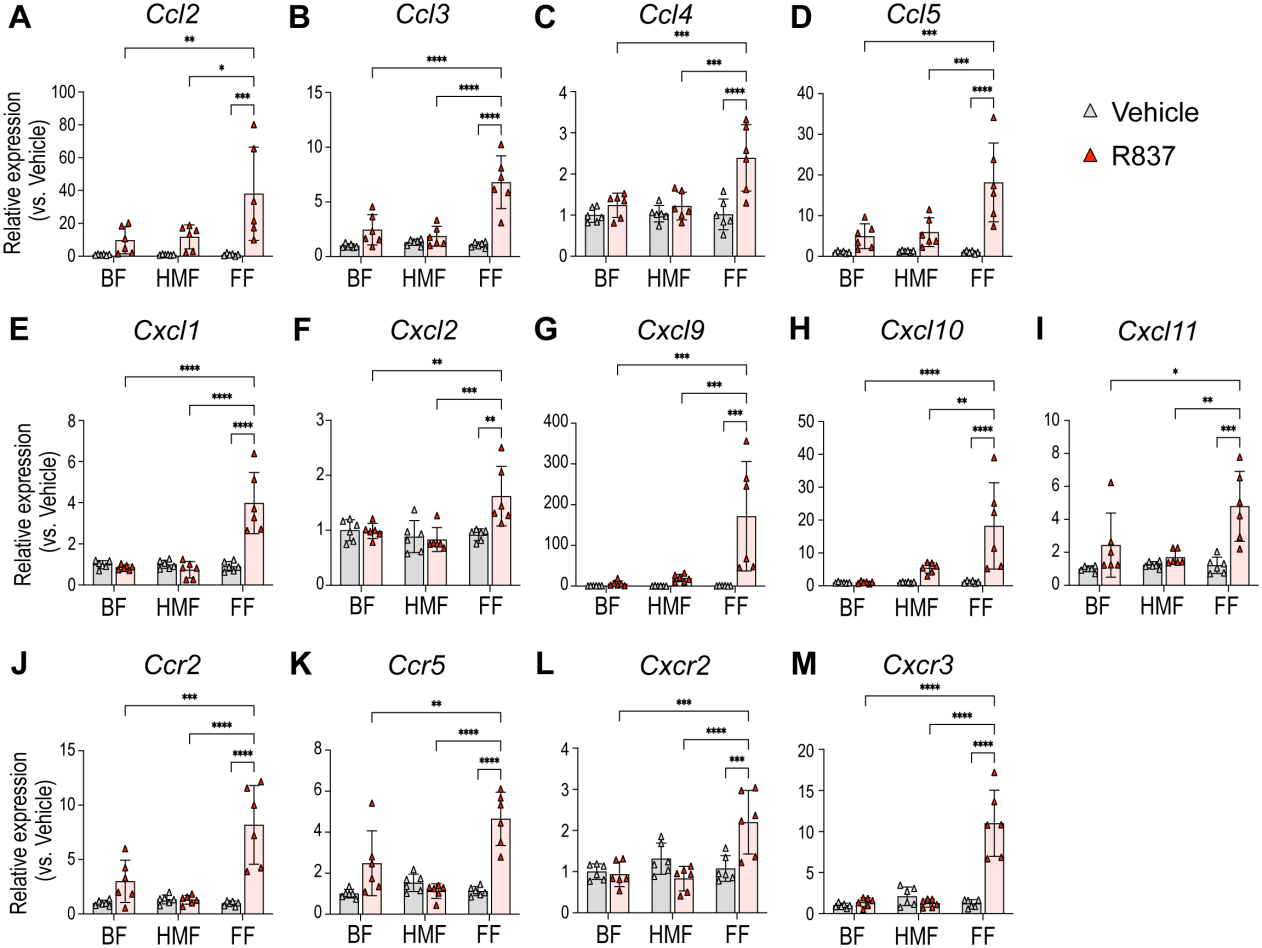
Formula feeding serves as a first hit-like priming factor that contributes to enhancing chemokine and chemokine receptor expression following a viral inflammatory second hit. Mouse pups (P7) from multiple litters were subjected to BF, HMF and FF for 4 days. In this experimental two-hit model, the feeding intervention constituted the priming first hit, followed by oral administration of the TLR7 agonist R837 as the inflammatory second hit. On P10, all mice were orally gavaged with R837 (25 μg/gm) or vehicle. Pups were euthanized at P11. **(A–M)** RT-qPCR analysis of the expression of chemokines and their receptors including *Ccl2* **(A)***, Ccl3* **(B)***, Ccl4***(C)***, Ccl5* **(D)***, Cxcl1* **(E)***, Cxcl2* **(F)***, Cxcl9* **(G)***, Cxcl10* **(H)***, Cxcl11* **(I)***, Ccr2* **(J)***, Ccr5* **(K)***, Cxcr2* **(L)** and *Cxcr3* **(M)** in the ileum of R837 or vehicle treated pups. PCR reactions were run in duplicate for each sample. Data are presented as means ± standard deviation; n = 6 in each group. Data represent two independent experiments and were analyzed by one-way ANOVA with Tukey posttest. *P < 0.05 was considered statistically significant. **P < 0.01, ***P < 0.001, ****P < 0.0001 (vs. BF).

**Figure 2:**
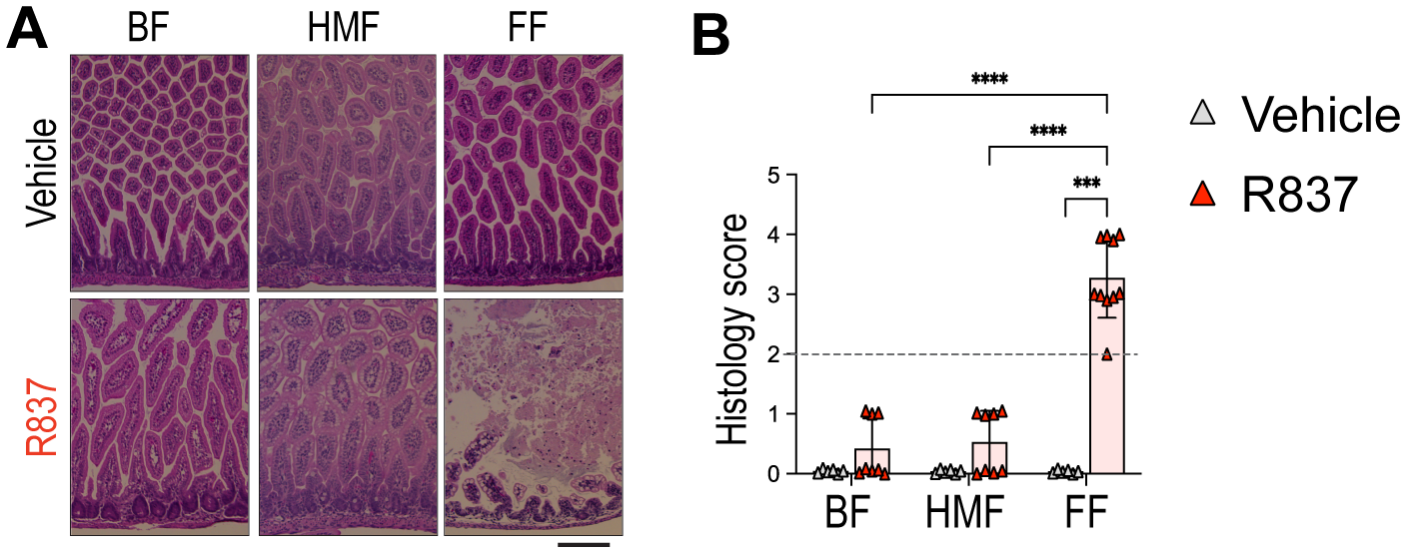
FF-associated first hit predisposes neonatal mice to NEC following an R837-induced inflammatory second hit. Mouse pups (P7) from multiple litters were subjected to BF, HMF and FF for 4 days. On P10, all mice were orally gavaged with R837 (25 μg/gm) or vehicle. **(A)** Representative microscopic images of hematoxylin-eosin (H&E) staining of the small intestinal sections from indicated experimental groups. Scale bar, 100 μm. **(B)** Quantification of histological scores of indicated experimental groups (n = 6-10/group). NEC-like injury was defined as histological grade ≥ 2. Histological scores were analyzed using the χ2 test. Data represent three independent experiments. ***P < 0.001, ****P < 0.0001.

For this purpose, we compared the gene expression levels of chemokines and their receptors (including *Ccl2, Ccl3, Ccl4, Ccl5, Cxcl1, Cxcl2, Cxcl9, Cxcl10, Cxcl11*, *Ccr2, Ccr5, Cxcr2*, and *Cxcr3*) in the ileum 24 h after R837-induced viral inflammation in neonatal pups subjected to FF by gavage from postnatal day 7 to 11 (P7-P11) with those of pups receiving breastmilk feeding (BF) or human donor milk-feeding (HMF) by gavage. Under basal conditions, the expression of these chemokines and their receptors was comparable among BF, HMF, and FF groups at P11 (**Figures 1A–M**), indicating that exposure to different feeding regimens did not induce widespread activation of chemokine-receptor signaling. These findings indicate that exposure to the FF-associated first hit alone was insufficient to trigger a pronounced inflammatory chemokine response. Strikingly, following the R837-induced second hit, only FF mouse pups, but not BF or HMF pups, exhibited a robust increase in the expression of chemokines, including *Ccl2, Ccl3, Ccl4, Ccl5, Cxcl1, Cxcl2, Cxcl9, Cxcl10*, and *Cxcl11*, in the small intestine (**Figures 1A–I**), indicating that FF primes the intestinal mucosa for an exaggerated inflammatory response following viral inflammation. Consistent with this observation, only R837-treated FF mouse pups displayed significant upregulation of the chemokine receptors *Ccr2, Ccr5, Cxcr2*, and *Cxcr3* (**Figures 1J–M**), further suggesting enhanced capacity for inflammatory-cell recruitment following sequential first-hit and second-hit exposure. In agreement with these molecular changes, NEC-like intestinal injury developed exclusively in FF pups within 24 h following R837 treatment (**Figure 2**). Collectively, our findings demonstrate that activation of chemokine-receptor signaling is closely associated with NEC development in the FF + R837 two-hit model. These findings establish chemokine-receptor activation as the inflammatory pathway investigated in the subsequent pharmacological inhibition studies.

### 3.2 Pharmacological blockade of CCR2/CCR5/CXCR3 signaling attenuates viral inflammation-triggered NEC in FF neonatal mice

Our recent findings demonstrated that viral inflammation-induced NEC in FF mouse pups is characterized by NK1.1^+^ cell-associated intestinal injury (21). Given the pivotal role of CCR2, CCR5, and CXCR3 in mediating recruitment of inflammatory cells, including NK cells (33–35), we investigated whether pharmacological inhibition of these receptors attenuates NEC development. TAK-779 is a synthetic, non-peptide CCR5 antagonist originally developed for HIV treatment (36). It has been reported that TAK-779 blocks ligand binding to multiple murine chemokine receptors, including CCR2, CCR5, and CXCR3 (28, 37–39) and reduces inflammation in experimental models of colitis and viral infection (28, 37). Thus, we hypothesized that blockade of CCR2/CCR5/CXCR3 signaling with TAK-779 suppresses inflammatory amplification and protects against NEC induced by sequential FF and viral inflammation in a two-hit setting.

**Figure 3:**
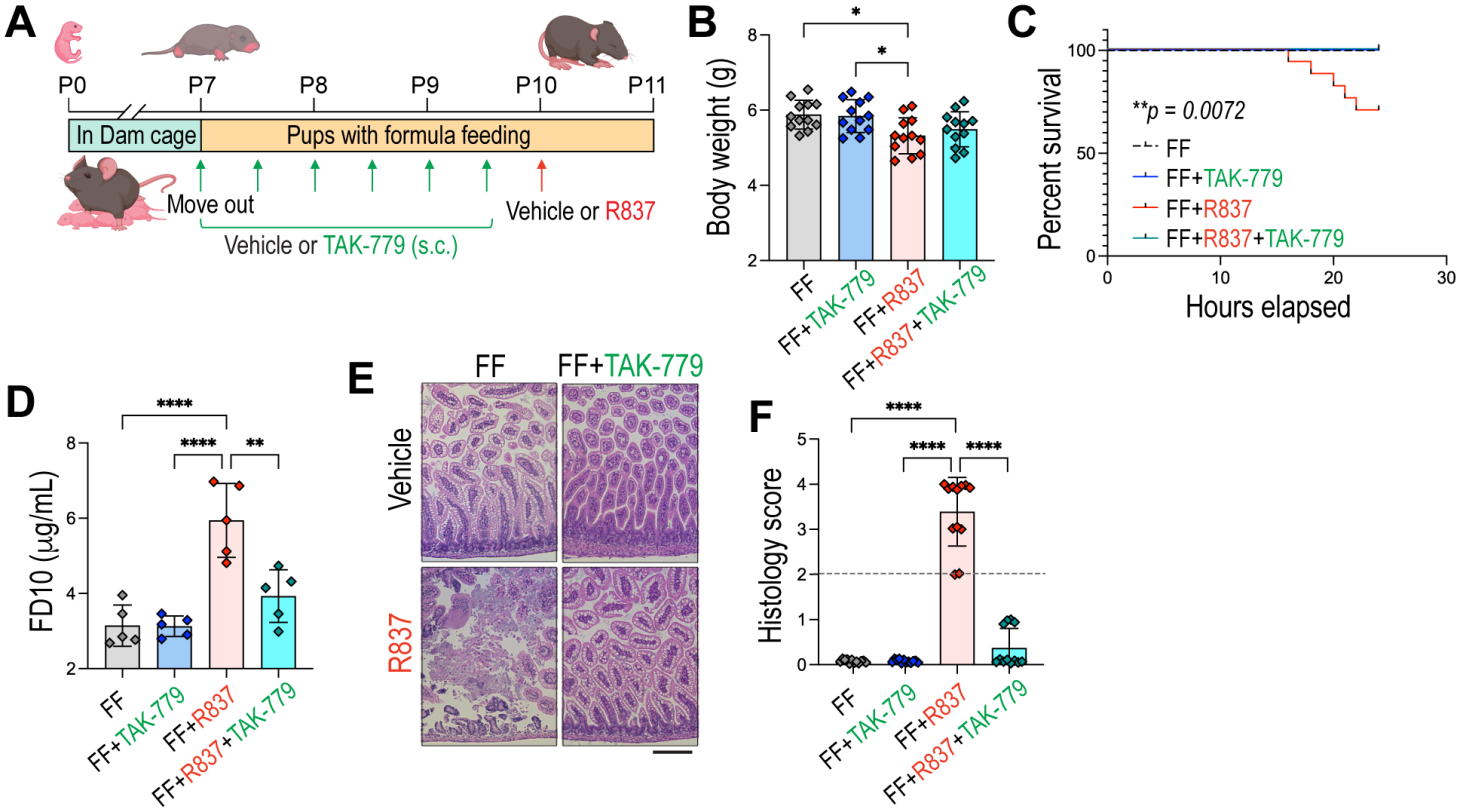
TAK-779 pretreatment attenuates two-hit-induced NEC in FF mouse pups. FF mouse pups (P7) were subcutaneously pretreated with the CCR2/CCR5/CXCR3 inhibitor TAK-779 (2.5 µg/gm, b.i.d) for 3 days from P7 – P10 to block CCR2, CCR5, and CXCR3 signaling. In this experimental two-hit model, FF functioned as a first hit, and R837 administration represented the inflammatory second hit. On P10, FF pups were treated with R837 (25 μg/gm, oral gavage) or vehicle. **(A)** Experimental design for FF + TAK-779 + R837 model. Panel was created with BioRender.com. **(B)** Body weight measurement at P11 of indicated experimental groups (n = 12/group). Data are presented as means ± standard deviation. Data represent three independent experiments and were analyzed by one-way ANOVA with Tukey posttest. *P < 0.05. **(C)** Analysis of the 24-h survival rate after indicated treatment (n = 12-17/group). Data represent three independent experiments and Log-rank test was used for survival curves. **P < 0.01. **(D)** relative levels of FD-10 in serum as a measure of intestinal permeability (n = 5/group). Intestinal permeability assay was run in duplicate for each sample. Data are presented as means ± standard deviation. Data represent two independent experiments and were analyzed by one-way ANOVA with Tukey posttest. **P < 0.01, ****P < 0.0001. **(E)** Representative microscopic images of hematoxylin-eosin (H&E) staining of the small intestinal sections from indicated experimental groups. Scale bar, 100 μm. **(F)** Quantification of histological scores of indicated experimental groups (n = 12/group). NEC-like injury was defined as histological grade ≥ 2. Histological score was analyzed by χ^2^ test. Data represent three independent experiments. ****P < 0.0001.

To test this notion, FF mouse pups were randomly assigned to appropriate experimental groups. There were no significant differences in body weight among experimental groups at the beginning of the experiment (P7) (**Supplementary Figure 1**). FF mouse pups were then subcutaneously pretreated with TAK-779 (2.5 µg/gm, b.i.d.) from P7-P10, followed by R837 administration at P10 for 24 h (**Figure 3A**). At the end of the experiment (P11), pretreatment with TAK-779 alone in FF mouse pups did not alter body weight, survival rate, or intestinal permeability compared with untreated FF cohorts (**Figures 3B–D**). After 24 h of R837 treatment, FF pups exhibited a marked reduction in body weight and survival rate, accompanied by a significant increase in intestinal permeability compared with FF alone and TAK-779-pretreated FF cohorts (**Figures 3B–D**). In contrast, all FF pups pretreated with TAK-779 survived after R837 challenge (**Figure 3C**). Moreover, intestinal barrier integrity was preserved in TAK-779-pretreated FF pups following R837 treatment compared with the FF + R837 group (**Figure 3D**). Histological examination revealed that FF pups pretreated with TAK-779 alone retained normal intestinal histological features comparable to FF cohorts (**Figures 3E, F**). Consistent with our prior findings, R837-treated FF pups developed segmental small intestinal injury within 24 h, characterized by villous necrosis, epithelial sloughing, and transmural necrosis, closely resembling human NEC pathology (**Figures 3E, F**). In contrast, TAK-779-pretreated pups in the FF + R837 cohort displayed only mild villus edema and superficial epithelial sloughing (**Figures 3E, F**), indicating that TAK-779 pretreatment markedly attenuated NEC development induced by R837 in FF pups. Collectively, these results demonstrate that pharmacological blockade of CCR2/CCR5/CXCR3 signaling confers robust protection against two-hit-induced NEC in FF neonatal mice, supporting a role for chemokine-receptor-dependent inflammatory responses in NEC pathogenesis.

### 3.3 TAK-779 attenuates the effect of FF combined with viral inflammation on the induction of chemokine-receptor expression and inflammatory cell recruitment

**Figure 4:**
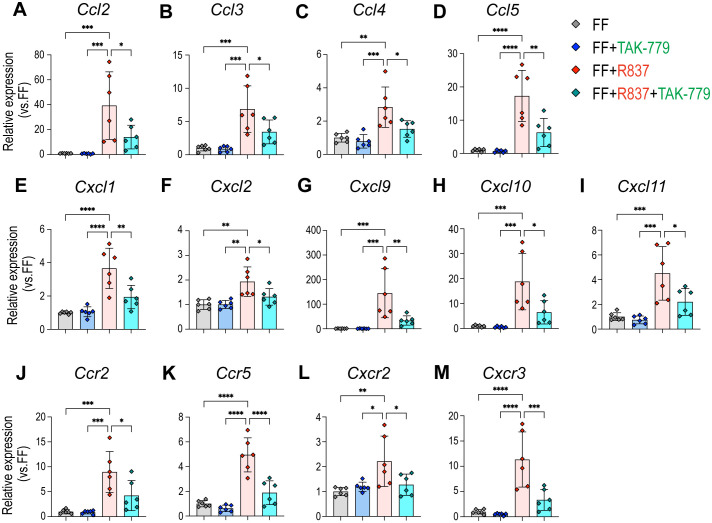
TAK-779 reduces chemokine and chemokine receptor expression during the two-hit process in FF mouse pups. FF mouse pups (P7) were subcutaneously pretreated with TAK-779 (2.5 µg/gm, b.i.d) for 3 days. On P10, FF pups were treated with R837 (25 μg/gm, gavage) or vehicle. **(A–M)** RT-qPCR analysis of the expression of chemokines and their receptors including *Ccl2* **(A)***, Ccl3* **(B)***, Ccl4* **(C)***, Ccl5* **(D)***, Cxcl1* **(E)***, Cxcl2* **(F)***, Cxcl9* **(G)***, Cxcl10* **(H)***, Cxcl11***(I)***, Ccr2* **(J)***, Ccr5* **(K)***, Cxcr2* **(L)** and *Cxcr3* **(M)** in the ileum of indicated experimental groups. PCR reactions were run in duplicate for each sample. Data are presented as means ± standard deviation; n = 6 in each group. Data represent two independent experiments and were analyzed by one-way ANOVA with Tukey posttest. *P < 0.05 was considered statistically significant. **P < 0.01, ***P < 0.001, ****P < 0.0001 (vs. FF).

Next, we sought to determine whether a sequential two-hit process consisting of FF followed by viral inflammation promotes NEC development through chemokine receptor-dependent inflammatory responses. To do so, FF neonatal mice were treated with TAK-779 prior to R837-induced viral inflammation, and chemokine-receptor expression was assessed by RT-qPCR. We found that the expression of chemokines and their receptors was comparable between FF-alone and TAK-779 pretreated FF groups (**Figures 4A–M**). As expected, administration of R837 robustly increased the expression of chemokines and their receptors in FF pups compared with FF-alone and TAK-779-pretreated FF groups (**Figures 4A–M**). In contrast, TAK-779 pretreatment markedly reduced the induction of chemokines, including *Ccl2, Ccl3, Ccl4, Ccl5, Cxcl1, Cxcl2, Cxcl9, Cxcl10*, and *Cxcl11* in the ileum of FF mouse pups following R837 exposure compared with FF + R837 pups (**Figures 4A–I**). Consistently, the expression levels of *Ccr2, Ccr5, Cxcr2*, and *Cxcr3* were markedly downregulated in the TAK-779-pretreated FF + R837 group (**Figures 4J–M**). These findings indicate that pharmacological blockade of CCR2/CCR5/CXCR3 signaling attenuates the induction of the chemokine-receptor axis triggered by viral inflammation in FF neonatal mice.

**Figure 5:**
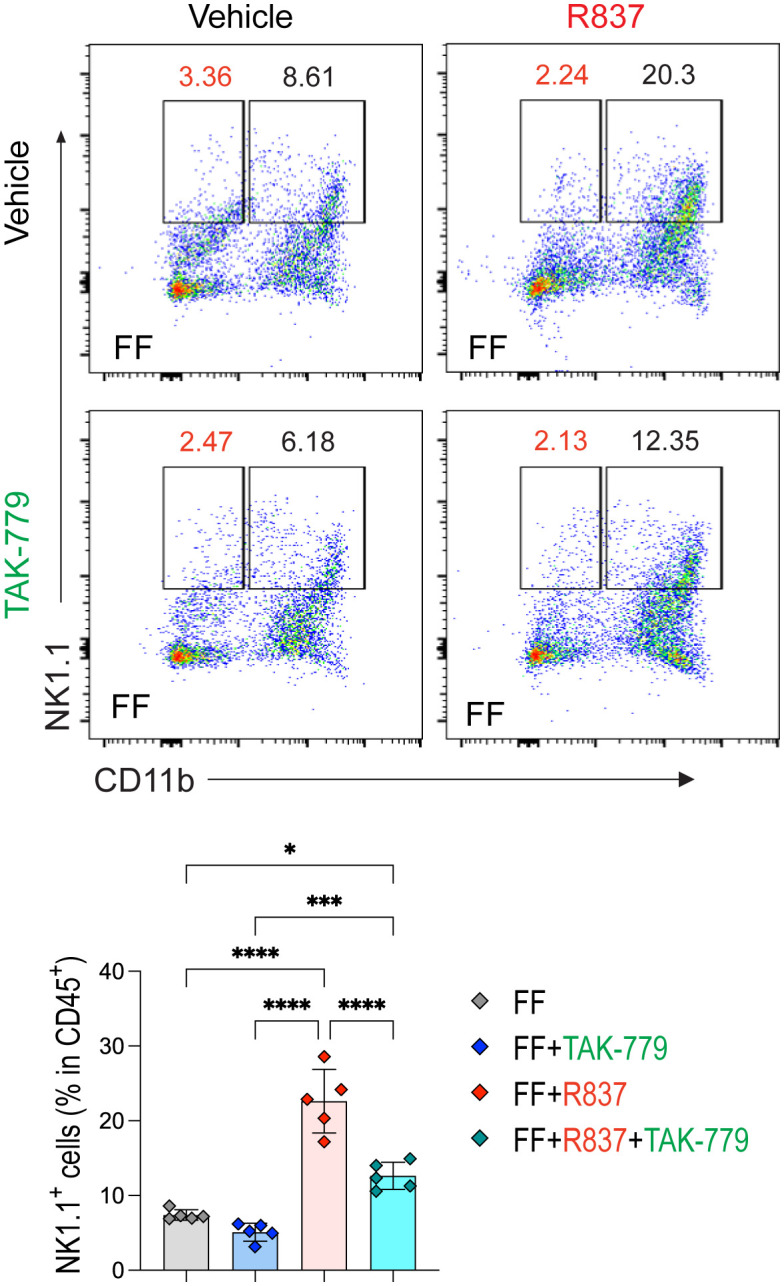
TAK-779 attenuates the infiltration of NK1.1^+^ cells during two-hit-induced intestinal inflammation in FF Mouse Pups. FF mouse pups (P7) were subcutaneously pretreated with TAK-779 (2.5 µg/gm, b.i.d) for 3 days. On P10, FF pups were treated with R837 (25 μg/gm, gavage) or vehicle for 24h. Representative flow cytometry and aggregate results for assessment of NK1.1^+^ cells (CD45^+^CD3^−^CD11b^+^NK1.1^+^) in the small intestine of indicated experimental groups. Data are presented as means ± standard deviation; n = 5 in each group. Data represent two independent experiments and were analyzed by one-way ANOVA with Tukey posttest. *P < 0.05 was considered statistically significant. ***P < 0.001, ****P < 0.0001.

**Figure 6:**
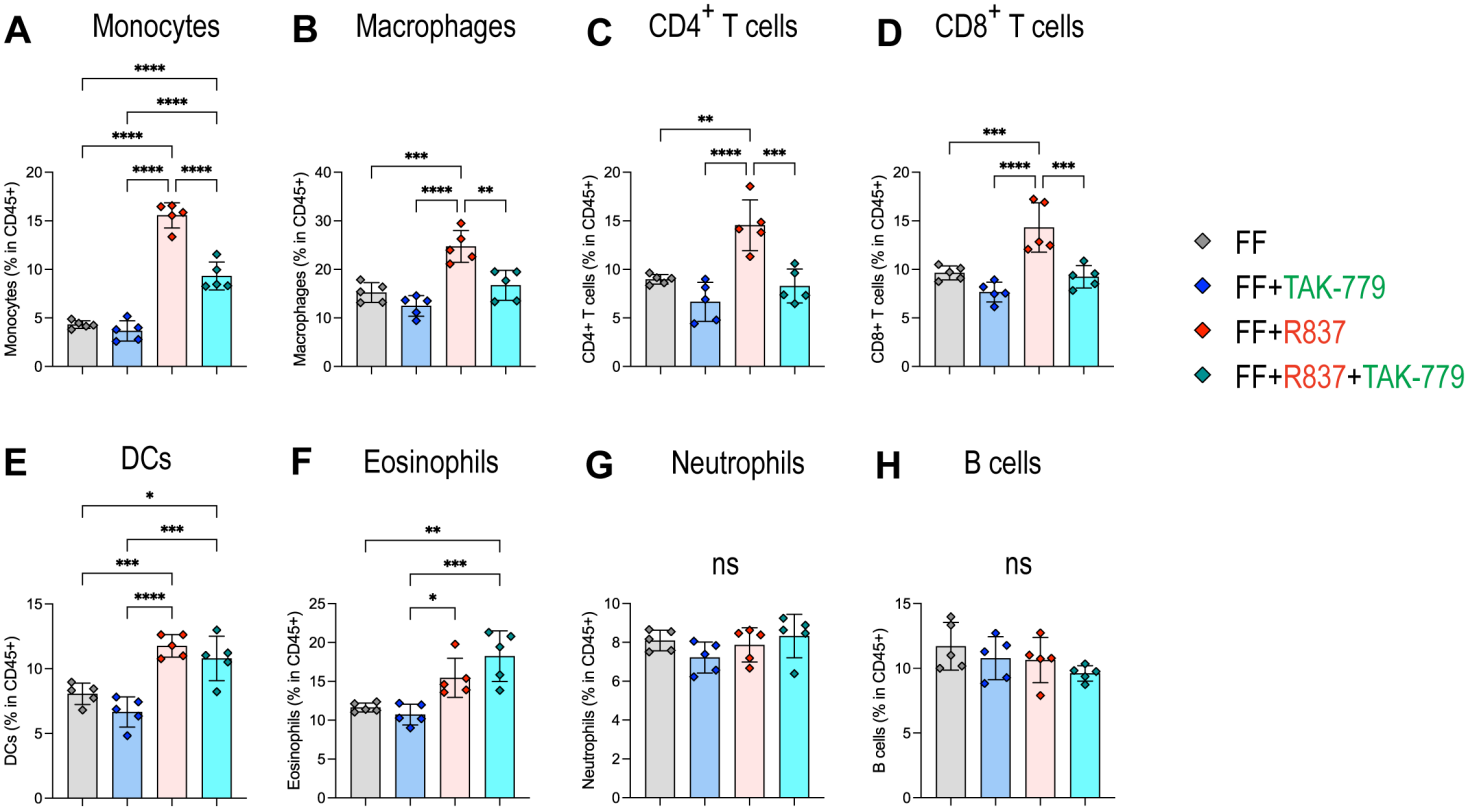
TAK-779 attenuates inflammatory cell recruitment in FF mouse pups during the two-hit process. FF mouse pups (P7) were subcutaneously pretreated with TAK-779 (2.5 µg/gm, b.i.d) for 3 days. On P10, FF pups were treated with R837 (25 μg/gm, gavage) or vehicle for 24h. **(A–H)** Flow cytometric analysis of infiltration of inflammatory cells including monocytes **(A)**, macrophages **(B)**, CD4^+^ T cells **(C)**, CD8^+^ T cells **(D)**, dendritic cells (DCs) **(E)**, neutrophils **(F)**, B cells **(G)**, and eosinophils **(H)** in the ileum of indicated experimental groups. Data are presented as means ± standard deviation; n = 5 in each group. Data represent two independent experiments and were analyzed by one-way ANOVA with Tukey posttest. *P < 0.05 was considered statistically significant. **P < 0.01, ***P < 0.001, ****P < 0.0001.

Furthermore, we assessed the infiltration of mucosal immune cells using flow cytometry (FACS), employing the gating strategy delineated in **Supplementary Figure 2**. FACS analysis revealed that TAK-779 pretreatment in FF pups resulted in frequencies of NK1.1^+^ cells (**Figure 5**) and other immune cell subsets that were comparable to those in FF-alone cohorts (**Figures 6A–H**). In agreement with our previous study (21), R837-treated FF pups displayed profound infiltration of inflammatory cells, including monocytes, macrophages, dendritic cells, CD4^+^ and CD8^+^ T cells, and NK1.1^+^ cells relative to FF controls (**Figures 5**, **6A–E**). Compared with TAK-779-pretreated FF cohorts, FF + R837 pups exhibited significantly higher proportions of monocytes, macrophages, dendritic cells, T cells, eosinophils, and NK1.1^+^ cells (**Figures 5**, **6A–F**). However, neutrophil and B-cell frequencies remained unchanged following R837 treatment (**Figures 6G, H**). Importantly, TAK-779 pretreatment markedly reduced NK1.1^+^ cell recruitment in R837-challenged FF pups compared with FF pups that did not receive TAK-779 pretreatment before R837 challenge (**Figure 5**). In parallel, TAK-779 also significantly attenuated the mucosal infiltration of monocytes, macrophages, CD4^+^ and CD8^+^ T cells in the ileum of FF pups subjected to viral inflammation (**Figures 6A–D**), indicating a broad dampening of mucosal inflammation. Notably, dendritic-cell frequencies were only minimally affected by TAK-779 treatment despite significant reductions in several other immune-cell populations (**Figure 6E**), suggesting differential chemokine dependencies among intestinal leukocyte subsets. Collectively, these findings show that CCR2/CCR5/CXCR3-associated chemokine signaling is a prominent inflammatory feature of NEC induced by the sequential FF- and viral inflammation-associated two-hit process. Pharmacological blockade of these pathways broadly attenuated inflammatory-cell recruitment, supporting the concept that TAK-779 protects against NEC by suppressing chemokine-receptor-dependent inflammatory responses in the neonatal gut.

### 3.4 Tilivalline-primed BF mouse pups display increased chemokine-receptor expression in the intestine and heightened susceptibility to NEC upon subsequent viral inflammation

Tilivalline (TV) is an enterotoxic metabolite produced by the pathobiont *Klebsiella oxytoca* species complex (KoSC) carrying *the til* biosynthetic gene cluster (22, 23, 40). Evidence has shown that NEC is frequently associated with increased colonization by KoSC harboring the *til* locus (24–26). Previously, we found that enteral administration of TV predisposed mouse pups to NEC triggered by viral inflammation (21). Because TV is a bacterial metabolite associated with FF that can function as a biologically relevant sensitizing first hit, we next examined whether TV similarly enhances chemokine-receptor-dependent inflammatory responses following a subsequent viral inflammation-derived second hit.

**Figure 7:**
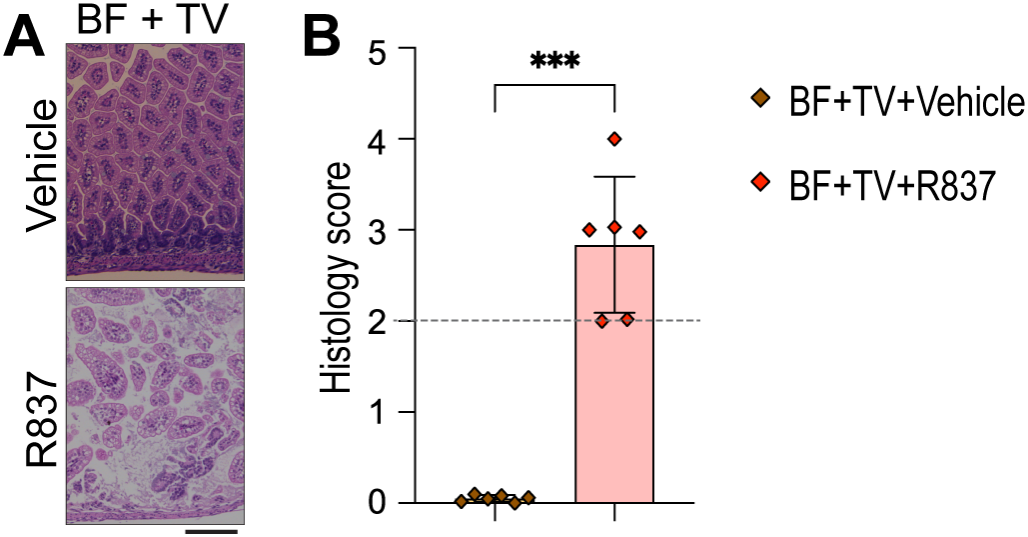
Tilivalline serves as a microbial first hit that predisposes BF mouse pups to R837-induced NEC. BF mouse pups (P7) were orally gavaged with TV (20 μg/pup/feeding, b.i.d) for 3 days to establish the first hit-induced priming. TV served as the microbial priming first hit before R837-induced viral inflammation as the second hit. On P10, BF mouse pups were treated with R837 (25 μg/gm, gavage) or vehicle for 24h. **(A)** Representative microscopic images of hematoxylin-eosin (H&E) staining of the small intestinal sections from indicated experimental groups. Scale bar, 100 μm. **(B)** Quantification of histological scores of indicated experimental groups (n = 6/group). NEC-like injury was defined as histological grade ≥ 2. Histological score was analyzed by χ2 test. Data represent three independent experiments. ***P < 0.001.

**Figure 8:**
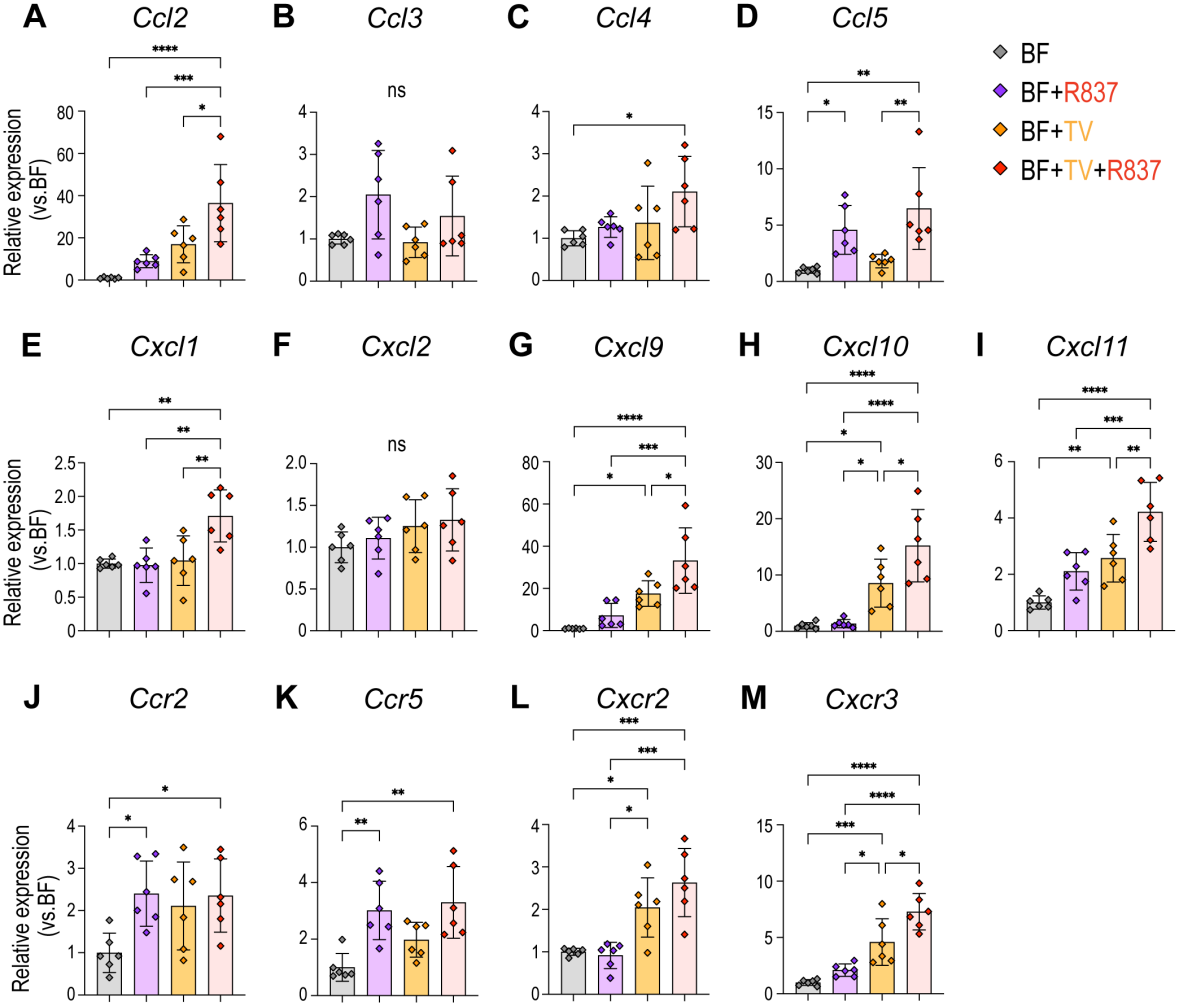
TV enhances chemokine and chemokine receptor expression during the two-hit process in BF mouse pups. BF mouse pups (P7) were orally gavaged with TV (20 μg/pup/feeding, b.i.d) for 3 days. TV constituted the microbial first hit followed by R837-induced viral inflammation as the second hit. On P10, BF mouse pups were treated with the TLR-7 agonist R837 (25 μg/gm, gavage) or vehicle for 24h. **(A–M)** RT-qPCR analysis of the expression of chemokines and their receptors including *Ccl2* **(A)***, Ccl3* **(B)***, Ccl4* **(C)***, Ccl5* **(D)***, Cxcl1* **(E)***, Cxcl2* **(F)***, Cxcl9* **(G)***, Cxcl10* **(H)**, *Cxcl11* **(I)**, *Ccr2* **(J)**, *Ccr5* **(K)**, *Cxcr2* **(L)** and *Cxcr3* **(M)** in the ileum of indicated experimental groups. PCR reactions were run in duplicate for each sample. Data are presented as means ± standard deviation; n = 6 in each group. Data represent two independent experiments and were analyzed by one-way ANOVA with Tukey posttest. *P < 0.05 was considered statistically significant. **P < 0.01, ***P < 0.001, ****P < 0.0001 (vs. BF). ns, not significant.

To test this end, BF mouse pups were orally gavaged with TV (20 μg/pup, b.i.d.) from P7-P10 and subsequently challenged with R837 at P10 for 24 h. Histological examination confirmed that this two-hit approach induced NEC in mouse pups (**Figure 7**). Meanwhile, compared with BF-alone cohorts, R837 administration significantly increased the expression of *Ccl5*, but not *Ccl2, Ccl3, Ccl4, Cxcl1, Cxcl2, Cxcl9, Cxcl10*, and *Cxcl11*, in the ileum of BF pups (**Figures 8A–I**). Correspondingly, the expression of chemokine receptors, including *Ccr2* and *Ccr5*, but not *Cxcr2* and *Cxcr3*, was upregulated in BF + R837 cohorts (**Figures 8J–M**). Interestingly, pretreatment with TV alone in BF mouse pups significantly increased the expression of *Cxcl9, Cxcl10*, and *Cxcl11*, without altering *Ccl2, Ccl3, Ccl4, Ccl5, Cxcl1*, and *Cxcl2* compared with BF cohorts (**Figures 8A–I**). This effect was accompanied by upregulation of *Cxcr2* and *Cxcr3*, but not *Ccr2* and *Ccr5*, in the ileum of BF + TV cohorts compared with the BF group (**Figures 8J–M**), suggesting that TV primes the neonatal intestinal mucosa for enhanced responsiveness through the CXCL9/10/11-CXCR3 axis. We also observed that *Cxcl10, Cxcr2*, and *Cxcr3* expression levels were significantly higher in BF + TV pups compared with BF + R837 pups (**Figures 8H, L, M**). Strikingly, TV-pretreated BF pups challenged with R837 exhibited a marked increase in the expression of *Ccl2, Ccl4, Ccl5, Cxcl1, Cxcl9, Cxcl10*, and *Cxcl11*, but not *Ccl3* or *Cxcl2*, when compared with BF controls (**Figures 8A–I**). Consistent with this chemokine amplification, *Ccr2, Ccr5, Cxcr2*, and *Cxcr3* expression levels were significantly upregulated in the ileum of BF pups receiving both TV and R837 (**Figures 8H–J**). Relative to BF + R837 pups, combined TV and R837 treatment in BF pups further increased *Ccl2, Cxcl1, Cxcl9, Cxcl10*, and *Cxcl11*, along with *Cxcr2* and *Cxcr3* expression (**Figures 8A, E, G–I, L, M**). Similarly, compared to BF + TV pups, BF pups receiving both TV and R837 exhibited increased *Ccl2, Ccl5, Cxcl1, Cxcl9, Cxcl10*, and *Cxcl11*, as well as *Cxcr3* expression (**Figures 8A, D, E, G–I, M**), indicating that TV priming enhances chemokine receptor-driven inflammatory responses during viral inflammation. Together, these data indicate that enhanced chemokine-receptor-dependent inflammatory responses are closely associated with NEC development in the TV + R837 two-hit model.

### 3.5 TAK-779 prevents viral inflammation-induced NEC in TV-primed BF mouse pups

**Figure 9:**
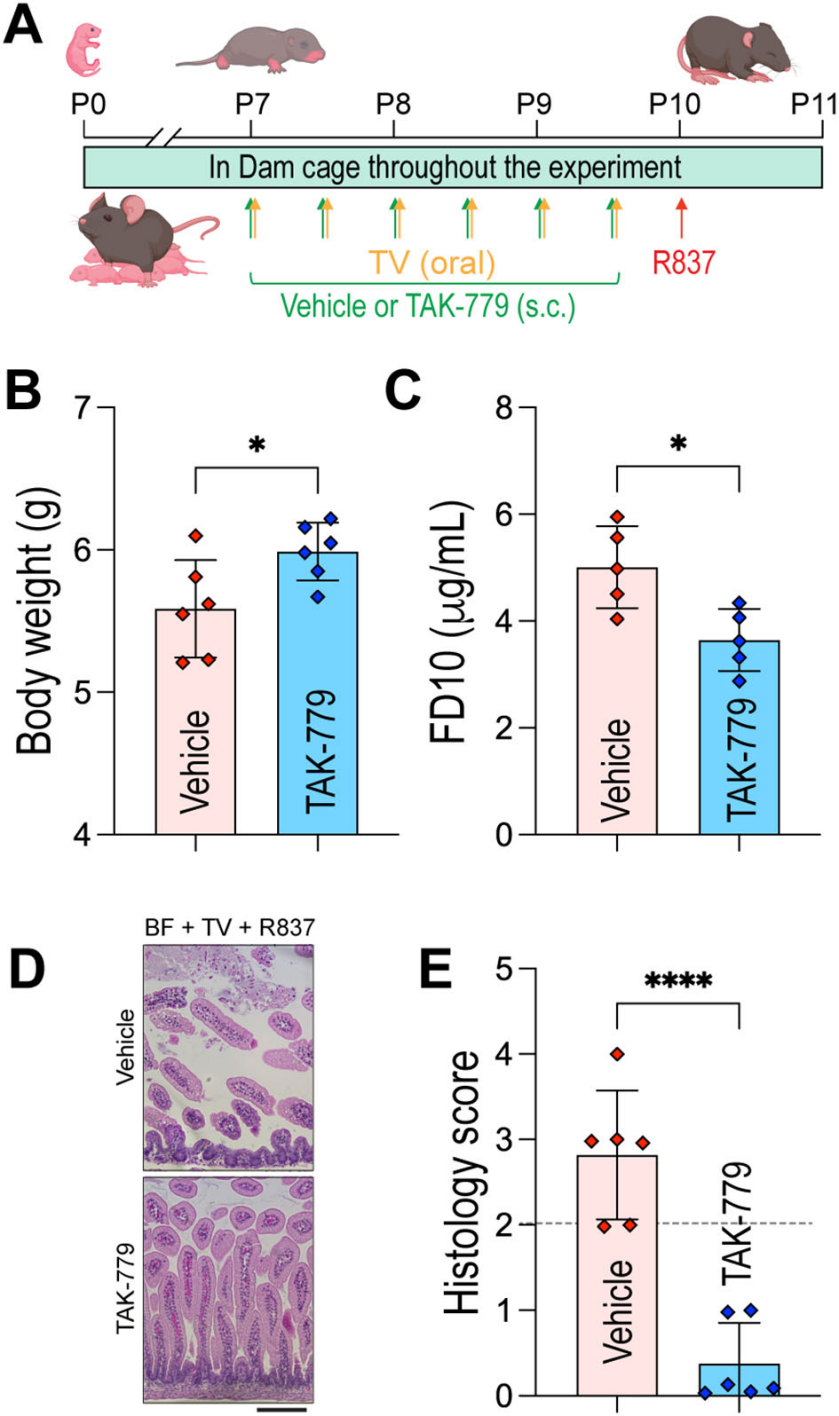
TAK-779 inhibits two-hit-induced NEC in TV-primed BF mouse pups. BF mouse pups (P7) were orally gavaged with TV (20 μg/pup/feeding, b.i.d) for 3 days. Simultaneously, these pups were subcutaneously pretreated with TAK-779 (2.5 µg/gm, b.i.d) or vehicle for 3 days. On P10, all mice were treated with R837 (25 μg/gm, gavage) for 24h. **(A)** Experimental design for BF + TV + TAK-779 + R837 model. Panel was created with BioRender.com. **(B)** Body weight measurement at P11 of indicated experimental groups (n = 6/group). Data are presented as means ± standard deviation. Data represent two independent experiments and were analyzed by student’s t-test. *P < 0.05. **(C)** Relative levels of FD-10 in serum as a measure of intestinal permeability (n = 5/group). Intestinal permeability assay was run in duplicate for each sample. Data are presented as means ± standard deviation. Data represent two independent experiments and were analyzed by student’s t-test. *P < 0.05. **(D)** Representative microscopic images of hematoxylin-eosin (H&E) staining of the small intestinal sections from indicated experimental groups. Scale bar, 100 μm. **(E)** Quantification of histological scores of indicated experimental groups (n = 6/group). NEC-like injury was defined as histological grade ≥ 2. Histological score was analyzed by χ^2^ test. Data represent two independent experiments. ****P < 0.0001.

Given that TV exposure followed by viral inflammation enhanced chemokine-receptor signaling in the gut and induced NEC, we next investigated whether pharmacologic inhibition of these pathways attenuates disease development using the TV + R837 two-hit model. For this purpose, BF mouse pups were randomly assigned to appropriate experimental groups. No significant differences in body weight were observed among experimental groups at baseline (P7) (**Supplementary Figure 3**). BF mouse pups were then subcutaneously pretreated with TAK-779 (2.5 µg/gm, b.i.d.) and orally gavaged with TV (20 μg/pup, b.i.d.) from P7-P10, followed by R837 administration at P10 for 24 h (**Figure 9A**). At the end of the experiment (P11), TAK-779-pretreated BF pups exposed to TV and R837 displayed a significant increase in body weight and a marked reduction in intestinal permeability compared with vehicle-treated BF mouse pups subjected to TV and R837 challenge (**Figures 9B, C**). Histopathological analysis further revealed that TAK-779 prevented the development of NEC-like tissue injury in BF pups exposed to TV + R837, as evidenced by preservation of villus architecture and reduced epithelial injury (**Figures 9D, E**). These findings indicate that TAK-779 attenuates the effect of TV priming and subsequent viral inflammation on NEC development by dampening chemokine-receptor-driven inflammatory responses.

**Figure 10:**
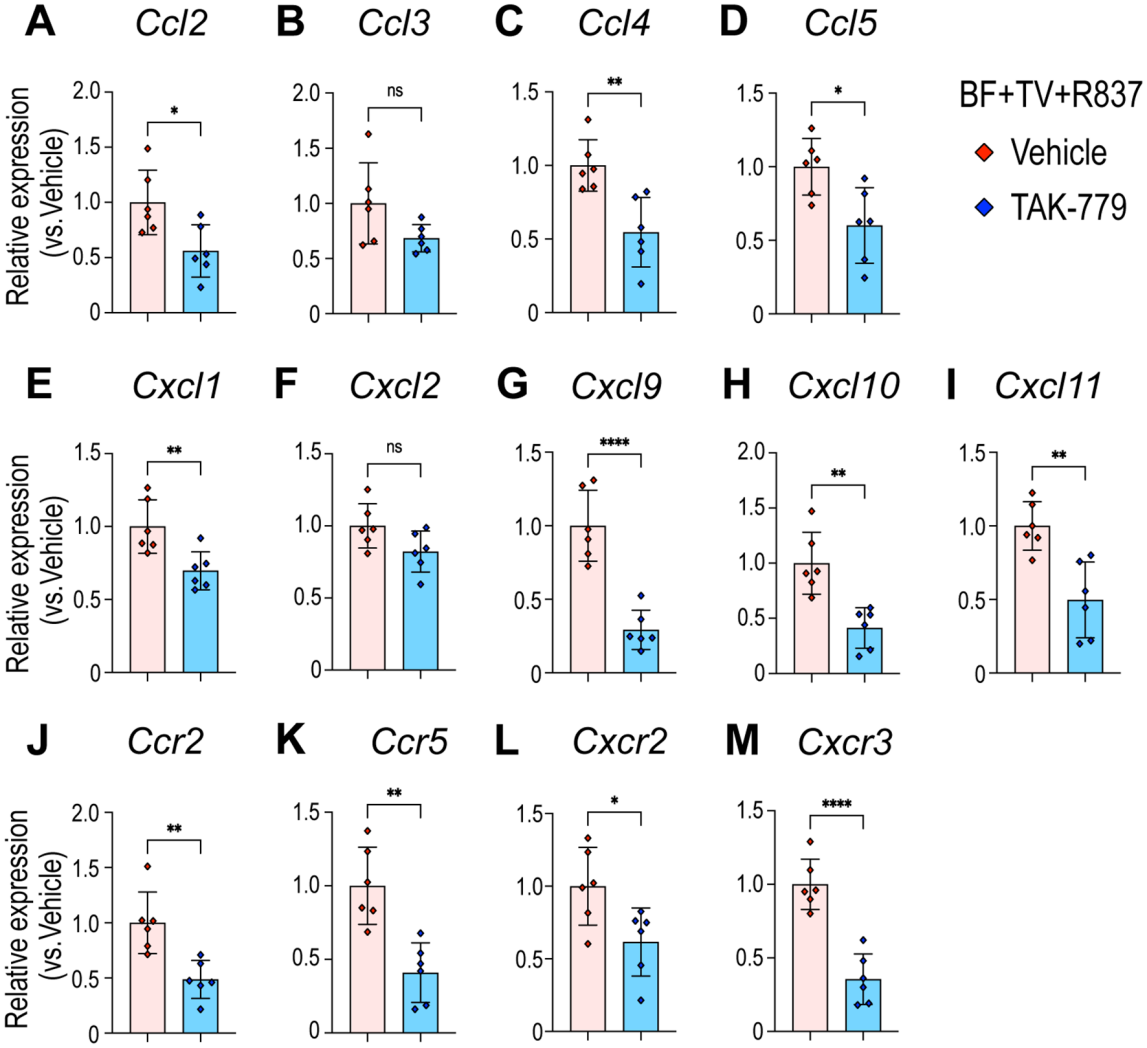
TAK-779 suppresses the effect of NEC-related two-hit on induction of chemokine and chemokine receptor expressions. BF mouse pups (P7) were orally gavaged with TV (20 μg/pup/feeding, b.i.d) for 3 days. At the same time, these pups were subcutaneously pretreated with TAK-779 (2.5 µg/gm, b.i.d) or vehicle for 3 days. On P10, all mice were treated with R837 (25 μg/gm, gavage). 24 h later, pups were euthanized and their ileum collected for RT-qPCR. **(A–M)** RT-qPCR analysis of the expression of chemokines and their receptors including *Ccl2* **(A)**, *Ccl3* **(B)**, *Ccl4* **(C)**, *Ccl5* **(D)**, *Cxcl1* **(E)**, *Cxcl2* **(F)**, *Cxcl9* **(G)**, *Cxcl10* **(H)**, *Cxcl11* **(I)**, *Ccr2* **(J)**, *Ccr5* **(K)**, *Cxcr2* **(L)** and *Cxcr3* **(M)** of indicated experimental groups. PCR reactions were run in duplicate for each sample. Data are presented as means ± standard deviation; n = 6 in each group. Data represent two independent experiments and were analyzed by student’s t-test. *P < 0.05 was considered statistically significant. ns, not significant. **P < 0.01, ****P < 0.0001 (vs. Vehicle). ns, not significant.

We next assessed the impact of TAK-779 on ileal chemokine and chemokine receptor expression in BF mouse pups challenged with TV + R837. Compared with BF pups treated with TV + R837, the expression levels of chemokines, including *Ccl2*, *Ccl4, Ccl5, Cxcl1, Cxcl9, Cxcl10, and Cxcl11*, but not *Ccl3* and *Cxcl2*, were significantly downregulated in the ileum of TAK-779-pretreated BF pups following TV + R837 challenge (**Figures 10A–I**). Consistently, TAK-779 pretreatment markedly reduced the expression of *Ccr2, Ccr5*, *Cxcr2*, and *Cxcr3* in the ileum of BF pups following TV + R837 challenge (**Figures 10J–M**). Together, these findings demonstrate that pharmacological inhibition of CCR2/CCR5/CXCR3 signaling effectively prevents NEC induced by the sequential TV + R837 two-hit challenge, further supporting chemokine-receptor-dependent inflammatory responses as a therapeutically targetable pathway for NEC prevention and treatment.

### 3.6 Human NEC tissues exhibit increased expression of multiple chemokines and chemokine receptors in the gut

**Figure 11:**
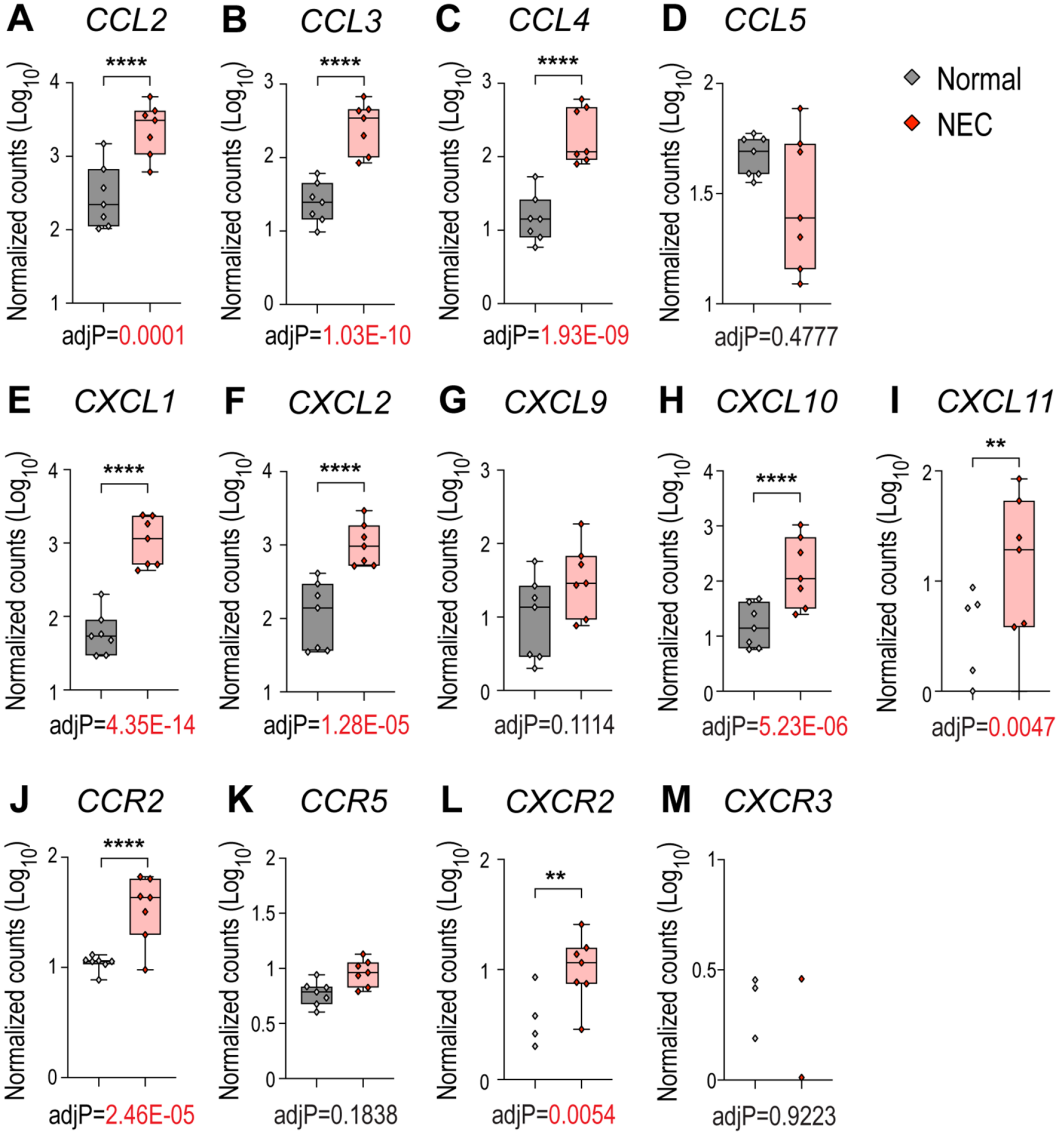
Meta-transcriptomic analysis reveals increased expression of chemokines and chemokine receptors in the intestines of patients with NEC. **(A–M)** Reanalysis of the published human RNA-seq dataset (E-MTAB-15683) showing the expression levels of chemokines and chemokine receptors signaling molecules including *CCL2* **(A)**, *CCL3* **(B)**, *CCL4* **(C)**, *CCL5* **(D)**, *CXCL1* **(E)**, *CXCL2* **(F)**, *CXCL9* **(G)**, *CXCL10* **(H)**, *CXCL11* **(I)**, *CCR2* **(J)**, *CCR5* **(K)**, *CXCR2* **(L)** and *CXCR3* **(M)** in intestinal tissues from patients with NEC and gestational age-matched controls (n = 7/group). Differentially expressed genes were identified using the DESeq2 package. *Adjusted (adj.)*P* < 0.05. **adj. *P* < 0.01; ****adj. *P* < 0.0001.

To determine the translational relevance of our experimental findings, we therefore reanalyzed a recently published human NEC RNA-seq dataset (29). This RNA-seq dataset was obtained from intestinal tissues of premature infants with surgical NEC (n = 7) and gestational age-matched controls with intestinal atresia (n = 7) (29). Differential gene expression (DEG) analysis revealed significantly increased expression of several chemokines (*CCL2*, *CCL3*, *CCL4*, *CXCL1, CXCL2*, *CXCL10*, and *CXCL11*) and chemokine receptors (*CCR2* and *CXCR2*) in NEC samples compared with controls (**Figures 11A–C, E, F, H–J, L**). CXCL9 and CCR5 expressions tended to be higher in NEC samples than in controls; however, these differences did not reach statistical significance (**Figures 11G, K**). No changes were observed in *CCL5* and *CXCR3* expression between the NEC and control groups (**Figures 11D, M**). Although the human dataset cannot establish the temporal sequence of the two-hit process, the shared chemokine-receptor signatures are consistent with inflammatory pathways identified in our experimental models. Collectively, these findings support the clinical relevance of chemokine-receptor-dependent inflammatory responses in NEC and strengthen the translational significance of our experimental observations.

## 4 Discussion

In this study, we identified a previously unrecognized role of chemokine-receptor-dependent inflammatory responses in NEC development induced by sequential bacterial and viral perturbations. Specifically, we used two novel and clinically relevant two-hit experimental NEC models that incorporate FF or TV followed by viral inflammation. We showed that FF and its associated microbial metabolite, TV, function as priming factors that sensitize the neonatal intestine to a subsequent viral inflammation (second hit), which is accompanied by activation of chemokine-receptor-dependent inflammatory responses and NEC onset. We demonstrate that FF-induced priming followed by viral inflammation selectively promotes the expression of chemokines and their receptors in the neonatal gut, particularly *Ccl2/3/4/5* and *Cxcl1/2/9/10/11*, along with their corresponding receptors (*Ccr2/Ccr5/Cxcr2/Cxcr3*). Remarkably, expression levels of these chemokines and receptors were also elevated in the intestines of human NEC subjects compared with controls. This NEC-related inflammation is associated with recruitment of inflammatory cells, including NK1.1^+^ cells in the small intestine. Moreover, we showed that TV, an NEC-associated bacteria-derived metabolite (21), functions as a potent modulator of this chemokine-receptor system. TV mimics the FF priming effect, thereby increasing intestinal susceptibility to NEC following a viral inflammation-derived second hit. Importantly, pharmacologic blockade of CCR2/CCR5/CXCR3 signaling with TAK-779 markedly attenuates inflammatory cell recruitment and protects neonatal mice from NEC in both two-hit experimental models. Collectively, these findings suggest that heightened chemokine-receptor-dependent inflammatory responses represent a key pathophysiological feature of two-hit-induced experimental NEC, thereby supporting further investigation of these pathways as promising therapeutic targets.

Chemokines are small heparin-binding proteins that orchestrate leukocyte migration through complex and disease-specific interactions with their receptors (30, 41). Substantial evidence shows that feeding mode profoundly influences neonatal gut immune homeostasis. Consistent with previous reports demonstrating that BF promotes immune tolerance and supports colonization by beneficial microbiota (42, 43), our data show that BF and HMF mouse pups maintain chemokine-receptor expression at homeostatic levels in the neonatal intestine during viral inflammation. In contrast, FF alone had little effect on chemokine-receptor expression, but markedly enhanced R837-induced expression of chemokines (*Ccl2, Ccl3, Ccl4, Ccl5, Cxcl1, Cxcl2, Cxcl9, Cxcl10*, and *Cxcl11*) and chemokine receptors (*Ccr2, Ccr5, Cxcr2*, and *Cxcr3*) in the neonatal intestine, as well as NEC development. These findings are consistent with the concept that FF acts as a priming first hit, whereas viral inflammation serves as the secondary hit that unmasks a heightened chemokine-receptor-dependent inflammatory response. Importantly, CCR2, CCR5, CXCR2, and CXCR3 are well-established mediators of immune cell recruitment during intestinal inflammation (41). Their respective ligand families, CCL2 for CCR2; CCL3, CCL4, and CCL5 for CCR5; CXCL1, and CXCL2 for CXCR2; and CXCL9, CXCL10, and CXCL11 for CXCR3, are widely expressed in inflamed intestinal tissues (32, 44–46). These chemokine axes facilitate recruitment of monocytes, macrophages, neutrophils, T cells, and NK cells during infection and tissue injury (31, 32, 44, 46). Thus, our findings suggest that the FF + R837-induced two-hit process triggers the expression of chemokines and chemokine receptors and generates a proinflammatory mucosal microenvironment that predisposes neonates to immune-mediated epithelial injury.

In clinical practice, FF of premature infants is commonly achieved through tube-assisted enteral feeding. Evidence shows that NEC-associated bacteria, such as *Klebsiella oxytoca*, are present in the biofilm of neonatal nasogastric enteral feeding tubes (19, 20). Colonization with these opportunistic pathogens profoundly alters gut microbial composition and metabolic outputs (17, 47, 48), thereby sensitizing the immature intestine to secondary inflammatory insults. This concept is further supported by emerging evidence demonstrating that bacterial metabolites act as immune cofactors that shape mucosal chemokine expression (49, 50). Previous studies have shown that *Klebsiella oxytoca* and its cytotoxin TV disrupt epithelial homeostasis, induce DNA damage, and promote inflammation (23, 51, 52). Our findings support this concept by demonstrating that TV functions as an FF-associated, bacteria-derived factor that contributes to NEC pathogenesis. In addition, we revealed that TV alone upregulated *Cxcl9/10/11-Cxcr3* expression but did not induce intestinal injury in BF pups. In contrast, sequential TV priming and R837-induced viral inflammation synergistically induced expression of *Ccl2, Ccl4*, and *Ccl5*, in addition to *Cxcl9, Cxcl10, and Cxcl11* expression. This effect was associated with NEC development. Collectively, we speculate that upregulation of chemokines and chemokine receptors is a critical pathophysiological feature of NEC induced by the sequential microbial metabolite- and viral inflammation-associated two-hit process.

A key finding of this study is that TAK-779 exerts a potent protective effect against NEC development. TAK-779 is a pharmacologic inhibitor that targets chemokine receptor signaling. We showed that TAK-779 pretreatment significantly improved survival, decreased intestinal permeability, attenuated histological injury, and prevented NEC development. These results provide evidence that CCR2/CCR5/CXCR3-dependent chemokine signaling is associated with inflammatory amplification during NEC development and that pharmacologic inhibition of these pathways attenuates disease severity in experimental models. Because TAK-779 was administered before viral challenge, our findings suggest that chemokine-receptor signaling contributes to amplification of the inflammatory response following the second hit rather than establishing that it is the initiating event in NEC pathogenesis. In addition, our results are consistent with previous studies showing that inhibition or deletion of CCR2/CCR5/CXCR3 mitigates intestinal inflammation and epithelial injury (28, 30–32, 46, 53). Notably, the robust protective effects of TAK-779 observed in these clinically relevant two-hit NEC models underscore the therapeutic potential of targeting chemokine-receptor-dependent inflammatory signaling as a strategy for preventing NEC.

We previously demonstrated that FF combined with viral inflammation induces NEC in an NK1.1**^+^** cell-dependent manner (21). We further demonstrated increased accumulation of NK1.1^+^ cells in experimental NEC and increased CD56^+^ cells in human NEC intestinal tissues by immunofluorescence, providing strong evidence supporting NK-cell involvement in NEC pathogenesis (21). In the current study, flow cytometric analyses demonstrated that TAK-779 reduced infiltration of NK1.1^+^ cells, monocytes, macrophages, and T lymphocytes into the neonatal intestine following viral challenge in FF pups. These findings support a strong association between coordinated chemokine-receptor signaling and inflammatory cell recruitment, including NK1.1**^+^** cells, during NEC onset. NK cells act as primary amplifiers of mucosal inflammation, initiating epithelial cytotoxicity and cytokine cascades (54, 55). In mucosal environments, NK cells serve as frontline sentinels, bridging innate and adaptive immunity, producing IFN-γ, and exerting cytotoxic functions against infected or stressed epithelial cells (56). During viral and inflammatory stress, upregulation of CCR5 ligands (CCL3-5), CXCR2 ligands (CXCL1-2), and CXCR3 ligands (CXCL9-11) enhances NK cell trafficking to infected tissues (33, 34, 41). Together, our current data and previous findings suggest that chemokine-receptor signaling promotes the recruitment of NK1.1^+^ cells and other inflammatory-cell populations, thereby contributing to NEC development under conditions of FF-associated priming followed by viral inflammation.

In addition, the attenuation of multiple inflammatory cell populations following TAK-779 treatment suggests that the protective effects observed in this study likely reflect suppression of a broader chemokine-receptor-dependent inflammatory network rather than selective inhibition of NK1.1^+^ cell recruitment alone. Importantly, because TAK-779 simultaneously targets CCR2, CCR5, and CXCR3, the present study does not distinguish the relative contributions of individual chemokine receptors. Among the receptors evaluated, CXCR3 was the most consistently induced receptor across both FF- and TV-associated models, suggesting that the CXCL9/CXCL10/CXCL11–CXCR3 axis may be an important pathway for future mechanistic investigation. CCR5 is also a plausible contributor because its ligands CCL3, CCL4, and CCL5 were robustly elevated in FF pups and are known mediators of NK1.1**^+^** cell and T-cell trafficking. CCR2 may contribute through recruitment of inflammatory monocytes and macrophages, which were also reduced following TAK-779 treatment. Therefore, future studies utilizing receptor-specific antagonists and genetic knockout models will be necessary to determine the relative importance of individual pathways in NEC pathogenesis.

An additional observation worth noting is the limited effect of TAK-779 on dendritic cell accumulation. We found that dendritic cell frequencies remained largely unchanged after TAK-779 treatment in the FF + R837 two-hit model. This finding suggests that dendritic cell recruitment in the neonatal intestine may rely on distinct chemokine pathways that are less dependent on CCR2, CCR5, or CXCR3 signaling. Although not the primary focus of the current study, these results reveal selective chemokine dependencies among immune-cell subsets and underscore the complexity of inflammatory cell trafficking in the neonatal intestine. Future studies investigating dendritic-cell-specific recruitment pathways may provide additional insight into immune regulation during NEC.

The translational relevance of our findings is supported by the reanalysis of a recently published human intestinal RNA-seq dataset (29). Our analysis revealed that human NEC tissues exhibited increased expression of several chemokines and chemokine receptors that overlapped with those identified in our experimental models. Notably, the overlap between the mouse and human datasets was incomplete. Several factors may contribute to these differences. First, human NEC specimens are inherently heterogeneous and may differ with respect to disease stage, clinical interventions (including antibiotic treatment and supportive care), and other patient-specific variables that influence gene-expression profiles. Second, intestinal tissues obtained from NEC patients typically represent advanced disease stages, whereas murine samples were collected at defined experimental time points. Third, species-specific differences in immune development, epithelial biology, and chemokine regulation may influence transcriptional responses. Finally, the relatively modest sample size available in the human dataset may have limited statistical power to detect certain differentially expressed genes. Despite these differences, the identification of overlapping chemokine-receptor signatures across species provides supportive translational evidence that dysregulated chemokine signaling is relevant to human NEC pathogenesis. Therefore, the human transcriptomic analysis should be interpreted primarily as supportive translational evidence rather than as a direct validation of all chemokine signatures identified in the experimental models.

Our findings highlight chemokine-receptor blockade as a promising therapeutic strategy for NEC. Small-molecule CCR5 antagonists such as maraviroc, already approved for HIV infection, and CXCR3 inhibitors currently under development for autoimmune diseases could potentially be repurposed to attenuate neonatal gut inflammation, although safety and development considerations must be carefully evaluated in premature infants. In addition, the identification of TV-producing *Klebsiella oxytoca* strains as potential upstream drivers of chemokine induction suggests opportunities for microbiome-targeted interventions, including probiotics, human milk oligosaccharides, or bacteriophage-based approaches.

Several limitations should be acknowledged. (1) TAK-779 inhibits multiple chemokine receptors, limiting mechanistic resolution regarding individual receptor contributions. Therefore, the current study cannot determine whether CCR2, CCR5, CXCR3, or a combination of these pathways is primarily responsible for the observed protective effects. (2) For targeted RT-qPCR analyses, individual genes were analyzed using ANOVA or Student’s T-test as appropriate. Formal correction for multiple testing across the entire 13-gene panel was not applied because these analyses were hypothesis-driven and focused on predefined chemokine targets. Therefore, individual gene-expression findings should be interpreted cautiously and validated in future studies. (3) The study primarily relies on gene-expression analyses and pharmacologic intervention rather than receptor-specific genetic approaches. (4) Neonatal pups were derived from multiple independent litters and randomly allocated across experimental groups whenever possible; however, litter-specific effects cannot be completely excluded. (5) Sex was not determined in neonatal pups, precluding assessment of sex as a biological variable. We cannot exclude the possibility that sex-specific effects contributed to the observed outcomes. Future studies incorporating sex determination and stratified analyses will be important for establishing the generalizability of these findings. (6) Histological injury scores were assigned based on the most severely affected intestinal segment on each slide. While this approach captures the most clinically relevant focal NEC-like lesions and is commonly used in experimental NEC studies, it may emphasize peak injury rather than overall severity across the entire intestinal section. (7) Our findings were generated in viral inflammation-associated NEC-like models and may not fully reflect other forms of experimental NEC. (8) Although TV was the focus of the current study, additional microbial metabolites may contribute to chemokine-receptor activation and intestinal injury. (9) Given the limited sample size of the human cohort (n = 7/group), findings from this secondary analysis should be interpreted cautiously and considered hypothesis-generating.

Despite these limitations, the present study identifies a reproducible association between chemokine-receptor activation, inflammatory-cell recruitment, and NEC-like intestinal injury across two clinically relevant two-hit experimental models of NEC, together with supportive findings from a human NEC dataset. Together with our previous demonstration that NK1.1^+^ cells are required for disease development in these models (21), the current study extends these findings by identifying chemokine-receptor-dependent inflammatory responses as an upstream inflammatory pathway associated with inflammatory-cell recruitment during NEC development. These findings provide a strong foundation for future receptor-specific genetic studies aimed at defining the precise molecular mechanisms underlying NEC pathogenesis.

## 5 Conclusion

In summary, our study suggests that chemokine-receptor-dependent inflammatory responses contribute to the propagation of NEC development. Using two clinically relevant two-hit experimental models of NEC, we demonstrate that FF or the microbial metabolite TV functions as a priming first-hit factor that increases intestinal susceptibility to NEC upon a viral inflammation-derived second hit. We further demonstrate that this two-hit pathophysiological process is accompanied by activation of chemokine-receptor-dependent inflammatory responses and the development of NEC-like intestinal injury. Increased expression of chemokines and their receptors coincided with inflammatory cell recruitment, including accumulation of NK1.1^+^ cells, and the development of NEC. Pharmacological inhibition of CCR2/CCR5/CXCR3 signaling reduced inflammatory responses and attenuated disease severity in both experimental models. Collectively, these findings support the concept that chemokine-receptor signaling represents an important inflammatory pathway through which sequential pathogenic insults promote NEC pathogenesis. Although the specific contributions of individual chemokine receptors and immune-cell subsets require further investigation, these results suggest that chemokine-receptor-dependent inflammatory pathways play an important role in two-hit-induced NEC development and warrant further investigation in both experimental and clinical studies as potential therapeutic targets.

## Data Availability

The original contributions presented in the study are included in the article/**Supplementary Material**. Further inquiries can be directed to the corresponding authors.
