# Supplementary material for: Chemokine-receptor-dependent inflammatory responses contribute to two-hit-induced experimental necrotizing enterocolitis and represent a potential therapeutic target
Source: Front Immunol. 2026 Jul 15;17:1830078. doi: 10.3389/fimmu.2026.1830078 (PMC13414843; doi:10.3389/fimmu.2026.1830078)
Supplement: Supplementary file 2 [file DataSheet2.pdf]

Supplementary Figures:

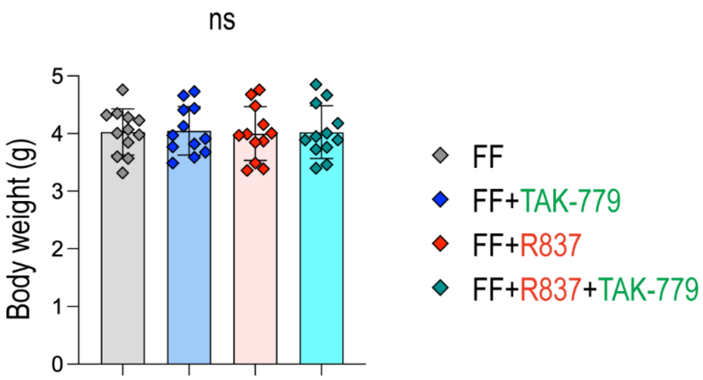

Supplementary Figure 1. Analysis of body weight at the start of the experiments for FF + TAK-779 + R837 intervention. Initial body weight measurement at P7 for indicated experimental groups (n = 12/group). Data are presented as means ± standard deviation. Data represent three independent experiments and were analyzed by one-way ANOVA with Tukey posttest. ns, not significant.

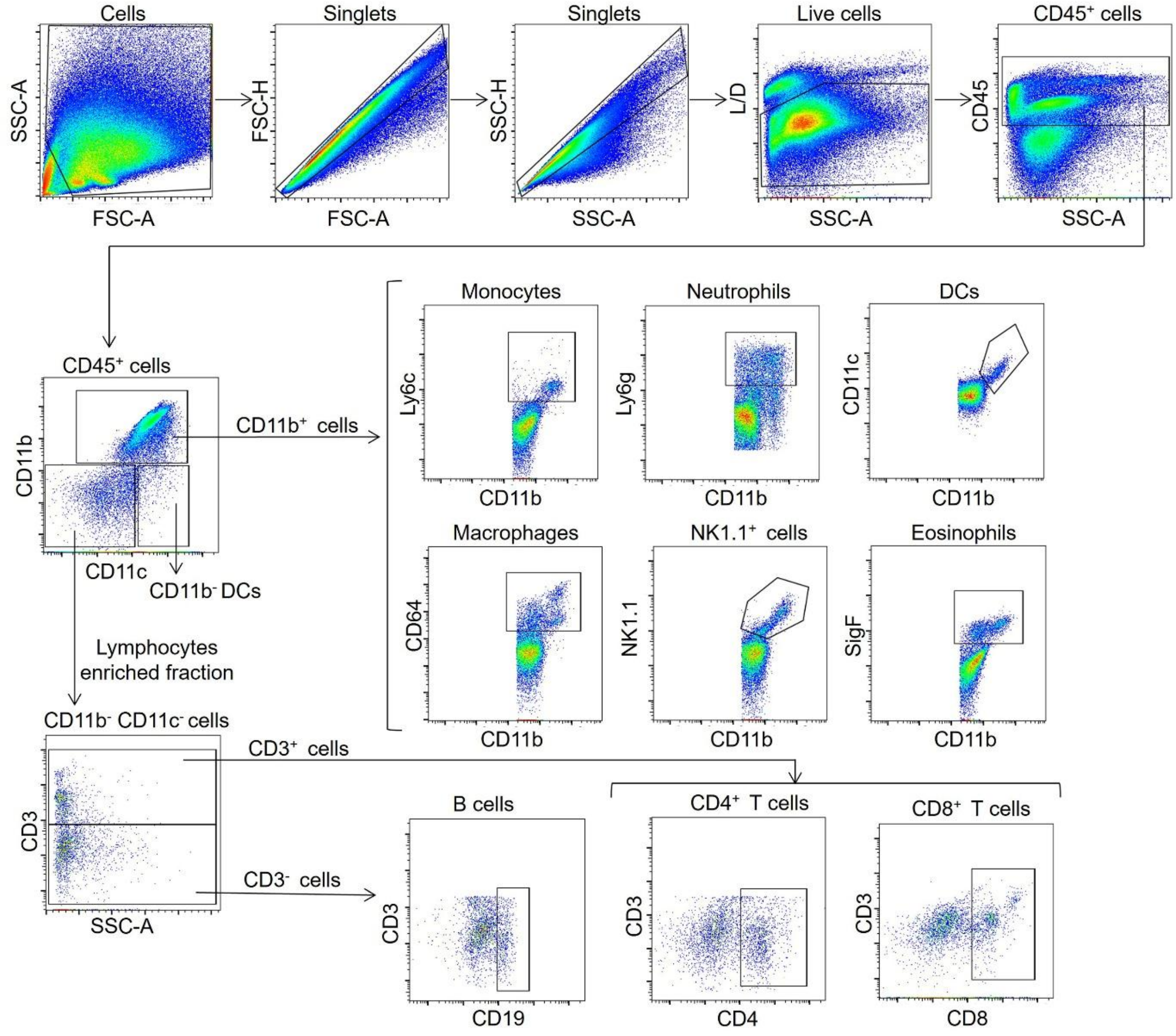

Supplementary Figure 2. FACS gating strategy for defining leukocyte subsets in the small intestine of mouse pups. To identify immune cell populations, debris and aggregates were first excluded using FSC-A vs. SSC-A profiles, followed by doublet discrimination via area versus height (FSC-A vs. FSC-H and SSC-A vs. SSC-H gates) comparisons. Live cells were isolated using a fixable viability dye (L/D), and hematopoietic lineage cells were identified by CD45 expression. From the CD45$^+$ fraction, CD11b$^+$ and CD11c$^+$ gates were applied to distinguish myeloid lineages: neutrophils (CD45$^+$CD3$^-$CD11b$^+$Ly6g$^+$), eosinophils (CD45$^+$CD3$^-$CD11b$^+$SigF$^+$), monocytes (CD45$^+$CD3$^-$CD11b$^+$Ly6c$^+$), macrophages (CD45$^+$CD3$^-$CD11b$^+$ Ly6G$^-$MHC-II$^+$CD64$^+$), dendritic cells (CD45$^+$CD3$^-$CD11b$^+$Ly6G$^-$CD64$^-$CD11c$^+$) and NK1.1$^+$ cells (CD45$^+$CD3$^-$CD11b$^+$NK1.1$^+$). Within the CD11b$^-$CD11c$^-$ lymphocyte-enriched gate, CD19$^+$ defined B cells (CD45$^+$CD11b$^-$CD3$^-$CD19$^+$), while T cell subsets were partitioned into CD4$^+$ T cells (CD45$^+$CD11b$^-$CD3$^+$CD19$^-$CD4$^+$) and CD8 T cells (CD45$^+$CD11b$^-$CD3$^+$CD19$^-$CD8$^+$) populations. All antibody concentrations were optimized through fluorescence-minus-one (FMO) titration.

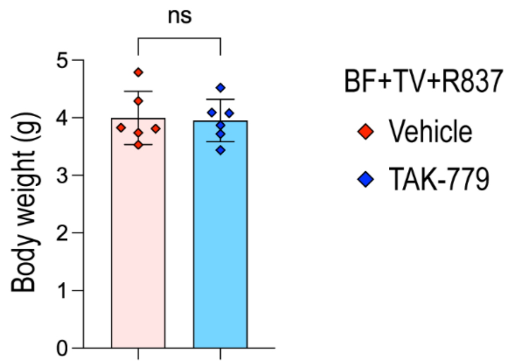

Supplementary Figure 3: Analysis of body weight at the start of the experiments for BF + TV + TAK-779 + R837 intervention. Initial body weight measurement at P7 for indicated experimental groups (n = 6/group). Data are presented as means ± standard deviation. Data represent two independent experiments and were analyzed by student's t-test. ns, not significant.
